# Phage mediated growth inhibition and biofilm disruption of the endodontic pathogen *Enterococcus faecalis*

**DOI:** 10.1371/journal.pone.0350657

**Published:** 2026-06-17

**Authors:** Daniel K. Arens, Meili Jensen, Meaghan A. Rose, Heather M. Zamora, Eun Y. Huh, Yoon Y. Hwang

**Affiliations:** Craniofacial Health and Restorative Medicine Directorate, Naval Medical Research Unit San Antonio, Joint Base San Antonio Fort Sam Houston, San Antonio, Texas, United States of America; The University of Texas Medical Branch at Galveston, UNITED STATES OF AMERICA

## Abstract

The failure of endodontic procedures such as root canal therapy is primarily indicated by persistent microbial infections. Root canal therapy involves the removal of decaying dental pulp (internal blood vessels and nerves of teeth), sanitizing the canal, and filling the space with biocompatible materials. Improper cleaning or the breakdown of these materials can lead to secondary infections. These infections, if left untreated, can lead to severe pain, bone and tooth loss, and potentially systemic infection. The bacterium *Enterococcus faecalis* is one of the most commonly associated organisms with failed root canal therapy, in addition to its prevalence in urinary tract infections, endocarditis, wounds, and sepsis. *E. faecalis* is known to survive in low nutrient environments and produce extensive biofilms, making it difficult to eradicate. In addition to antibiotic treatment, bacteriophages (phages), which are bacteria-specific viruses that kill their host are an interesting companion or alternative to antibiotics. In this study we isolated and characterized 14 *E. faecalis* phages from wastewater samples by testing their host range, growth inhibition, and biofilm eradication capabilities against several *E. faecalis* strains including two that were orally derived. Several phages showed broad host ranges (up to 16 strains), strong bacterial growth inhibition even when applied at low concentrations, and significant eradication of mature biofilms (97% reduction). The phages presented here represent a unique repertoire of antibacterial agents for use in treating endodontic infections and add to the growing library of *E. faecalis* phages to treat diverse infections.

## Introduction

Endodontics is a specialized field within dentistry focused on diagnosing and treating conditions affecting the dental pulp, the nerves and blood vessels inside a tooth. The most common procedure performed by endodontists is root canal therapy (RCT), which involves removing the pulp, cleaning the canal, and sealing the space with biocompatible materials. In certain cases, such as when there is a large infection or a tooth has multiple roots, RCT may require multiple visits. According to the American Association of Endodontists, approximately 15 million root canals are performed every year in the United States [1]. Worldwide ~55% of people have undergone RCT for at least one tooth [2].

RCT is typically necessary when a tooth has sustained trauma or when the pulp becomes infected. Despite the precautions taken, approximately 7–35% of teeth treated with RCT fail, the factors behind these rates depend on patient age, oral health habits, condition of the initial infection, and treatment method, although the exact cause of failure is likely multifactorial [3–12]. Reinfection after failure is predicted to occur in approximately half of adults worldwide, making it a critical hurdle when teeth are retreated [13–16]. If reinfection occurs, management depends on its location. In some cases, an apicoectomy, removing and sealing the tip of the root, may be necessary, particularly when infection spreads to the bone and gingival tissue at the root apex.

The primary pathogen associated with secondary infections is *Enterococcus faecalis*. Although not a typical resident of the oral microbiome [17], *E. faecalis* has been consistently identified in infected root canal samples through both culture-based methods and metagenomic studies [18–23]. It has been suggested that *E. faecalis* is transiently introduced into the mouth [17], allowing for secondary infections to occur weeks, months, or years later depending on the condition of the root canal. Its persistence is attributed to its ability to survive in harsh, nutrient-depleted environments, such as those created after treatment [24]. A major contributor to its persistence is its capacity to form biofilms [24,25].

Given the resilience of *E. faecalis* and the limited role of antibiotics in RCT [26–28], novel disinfection strategies are urgently required. Bacteriophages (phages), viruses that specifically infect and lyse bacteria, represent a potential adjunct therapy. Phages have been investigated extensively for diverse clinical applications [29–31], and *E. faecalis*-specific phages have been isolated for the treatment of urinary tract infections [32], bacteremia [33], wound infections [34], and endodontic infections [35]. Biofilm formation is central to these infections, as *E. faecalis* readily adheres to urinary catheters [36], cardiac implants [37], and natural tooth structures [17]. In dentistry, multiple research groups have explored the use of phages to target *E. faecalis* and evaluated their anti-biofilm activity [35,38–40]. Nonetheless, their therapeutic potential in endodontics remains underexplored.

In a previous Microbiology Resource Announcement [41], we reported the isolation of 14 taxonomically diverse *E. faecalis* phage that fell into three known viral genera including *Efquatrovirus* (Haystack, LoneStar, Alamo, Stockyards, RioGrande, TexasRanger, PricklyPear, Vaquero, TwoStep), *Saphexavirus* (GiddyUp, Pumpjack), *Kochikohdavirus* (Riverwalk, AllMyExes), and the proposed *Revolvervirus* (Revolver), which was genomically unique and may constitute a new genus (pending further review).

The work herein describes the morphology, host range, efficiency of plating, bacterial growth curve inhibition, and mature biofilm disruption of these phage. Moreover, this study addresses the gap in endodontic infection prevention strategies by isolating *E. faecalis*-specific phages for use as adjuncts to conventional RCT, with the aim of improving disinfection efficacy and reducing the incidence of secondary infections.

## Results

### Lysogeny, virulence, antimicrobial resistance gene detection

Manual inspection of phage genomes did not reveal any genes associated with a temperate lifestyle or virulence, or antimicrobial resistance. PhageAI [42] predicted that all 14 phages have lytic lifestyles. PhageLeads [43] confirmed the manual inspection with one exception in GiddyUp where it identified a hypothetical protein (XUJ69615) that could be associated with a temperate lifestyle. This protein is 57 amino acids long and is also annotated in another phage, SDS2 (ON113172), with a two amino acid difference (UQT00904). When SDS2 was analyzed using PhageLeads, no temperate lifestyle genes were detected.

### Transmission electron microscopy

**Fig 1:**
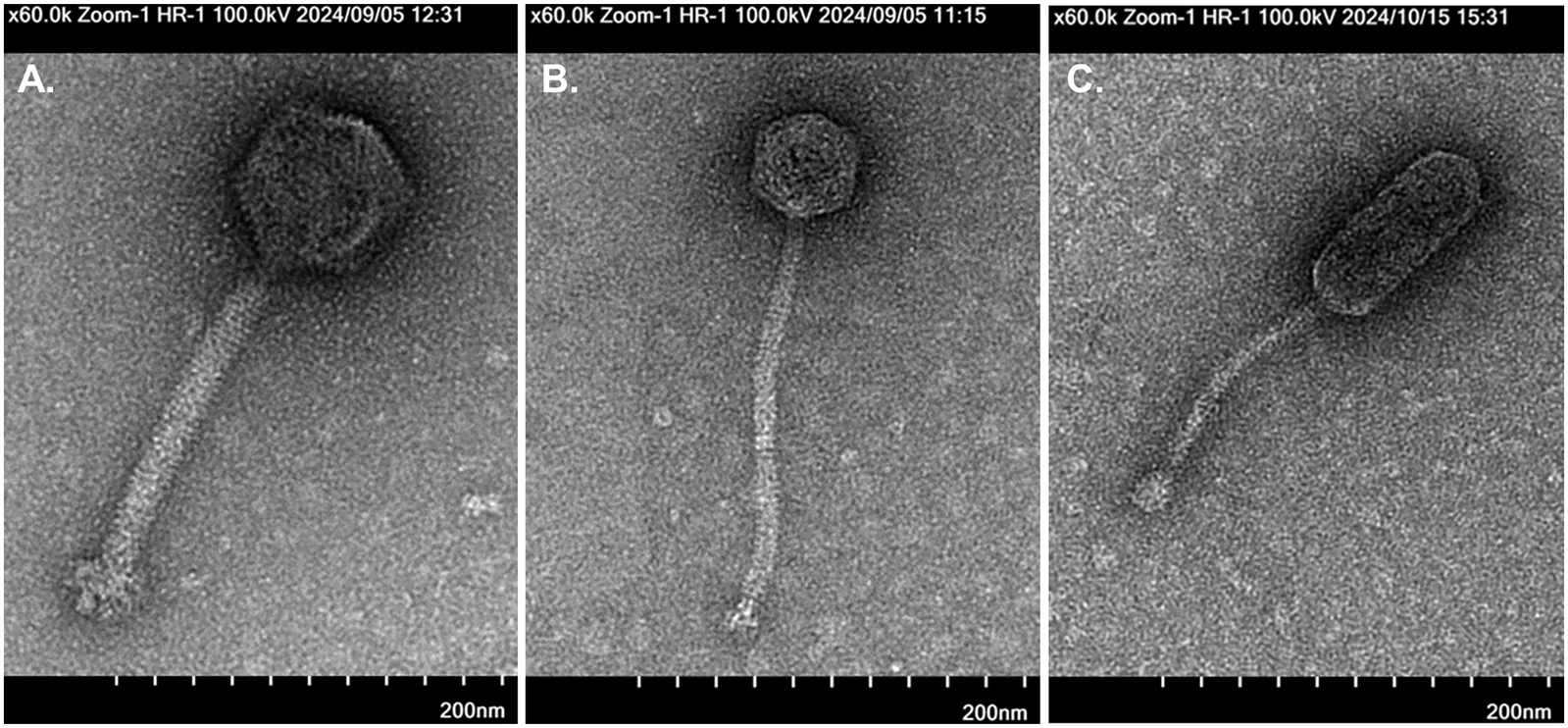
Transmission electron microscopy images of (A) Revolver, (B) Alamo, and (C) GiddyUp. Imaging was performed at the Keith R. Porter Imaging Facility at the University of Maryland, Baltimore County by Dr. Tagide deCarvalho.

TEM imaging revealed that the phage fall into two morphological groups: myovirus and siphovirus. The myoviruses include AllMyExes, Riverwalk, and Revolver. The siphoviruses include Alamo, LoneStar, TwoStep, Vaquero, PricklyPear, TexasRanger, Haystack, RioGrande, Stockyards, Pumpjack, and GiddyUp the *Efquatrovirus* and *Saphexavirus* phages. Pumpjack and GiddyUp are unique among this set of phage for having prolate heads. Among the remainder of the phages, the myoviruses had larger heads and shorter but wider tails than the siphoviruses. Head and tail dimensions are summarized in Table 1 and individual replicate measurements are provided in S1 File. Representative images of the three morphologies (Revolver, Alamo, and GiddyUp) are shown in Fig 1A, 1B, 1C. TEM images of the remaining 11 phage are provided in S1A–S1K Figs.

**Table 1:** Phage morphology measurements.

	AllMyExes	Riverwalk	Revolver	Alamo	LoneStar	TwoStep	Vaquero	PricklyPear	TexasRanger	Haystack	RioGrande	Stockyards	Pumpjack	GiddyUp
**Head Length (nm ± SD)**	**87.7 ± 2.3**	**93.8 ± 1.8**	**88.1 ± 2.4**	**53.4 ± 1.3**	**54.9 ± 0.3**	**54.4 ± 2.8**	**54.5 ± 2.0**	**55.1 ± 1.9**	**57.3 ± 2.0**	**55.4 ± 1.9**	**51.4 ± 4.1**	**55.1 ± 0.3**	**107.1 ± 3.7**	**107.0 ± 3.4**
**Head Width (nm ± SD)**	**79.4 ± 6.5**	**82.7 ± 3.3**	**79.0 ± 4.1**	**52.8 ± 2.1**	**49.8 ± 2.7**	**53.9 ± 4.0**	**51.6 ± 2.3**	**52.0 ± 1.5**	**51.1 ± 1.0**	**51.2 ± 1.7**	**49.3 ± 0.6**	**55.2 ± 1.5**	**42.1 ± 1.1**	**42.1 ± 0.2**
**Tail Length (nm ± SD)**	**169.7 ± 3.3**	**174.3 ± 2.4**	**170.1 ± 6.6**	**200.6 ± 5.8**	**200.3 ± 5.5**	**204.9 ± 1.7**	**205.8 ± 1.7**	**203.1 ± 3.6**	**200.6 ± 2.5**	**203.1 ± 3.3**	**200.4 ± 1.7**	**211.9 ± 4.1**	**132.1 ± 0.8**	**138.5 ± 7.0**
**Tail Width (nm ± SD)**	**19.8 ± 2.4**	**21.3 ± 1.3**	**19.1 ± 1.1**	**11.8 ± 0.7**	**11.4 ± 1.3**	**12.8 ± 1.2**	**11.8 ± 0.7**	**10.6 ± 0.9**	**12.3 ± 0.6**	**13.0 ± 0.4**	**10.1 ± 1.2**	**9.5 ± 2.7**	**10.1 ± 2.8**	**12.6 ± 0.5**

A minimum of three images were used for all measurements except for RioGrande with n = 2.

### Host range and efficiency of plating

The two kochikohdaviruses, AllMyExes and Riverwalk, along with the marginally related phage Revolver, had the broadest host ranges, infecting 9–16 of the 21 strains. All three phages were able to infect both orally derived strains. The *Efquatrovirus* phages had narrow host ranges with Alamo, LoneStar, TwoStep, and Vaquero infecting only their original host strains, 29212 or 33186. The remainder of the phages PricklyPear, TexasRanger, Haystack, RioGrande, and Stockyards only infected two or three strains, including the root canal strain 4082. No *Efquatrovirus* phage infected the other orally derived strain, OG1RF. Both saphexaviruses Pumpjack and GiddyUp, had narrow ranges with one or two hosts, respectively, including OG1RF. Neither were able to infect the root canal strain 4082. During initial host range experiments spot testing showed several phage capable of inhibiting bacterial growth to various degrees, as indicated by (+) signs in Table 2, but were unable to produce plaques during efficiency of plating testing. Plaque counts and efficiency of plating calculations can be found in S2 File.

**Table 2:** Phage host range & efficiency of plating.

Bacteria\Phage	AllMyExes	Riverwalk	Revolver	Alamo	LoneStar	TwoStep	Vaquero	PricklyPear	TexasRanger	Haystack	RioGrande	Stockyards	Pumpjack	GiddyUp
**4082**	**1.488**	**2.049**	**0.676**					**0.572**	**+**	**0.356**	**0.581**	**0.211**		
**OG1RF**	**2.500**	**1.496**	**0.944**										***1.000***	**2.384**
**29212**		***1.000***		***1.000***	***1.000***	***1.000***							**+**	***1.000***
**33186**	***1.000***	**1.680**	***1.000***				***1.000***	***1.000***	***1.000***	***1.000***			**+**	
**19433**	**1.402**	**2.871**	**0.829**					**9.15E-06**	**+**	**7.63E-06**	***1.000***	***1.000***		
**51299**	**1.113**	**1.457**	**0.572**					**+**		**+**	**+**	**+**		
**49624**			**+**					**+**						
**35667**			**+**					**+**	**+**	**+**				
**19434**			**+**					**+**	**+**	**+**				
**VRE01**		**0.535**	**+**					**+**	**+**	**+**	**+**	**+**		
**VRE02**	**1.032**	**1.085**	**0.446**					**+**	**+**		**+**	**+**		
**VRE05**		**0.551**	**+**					**+**	**+**	**+**	**+**	**+**		
**VRE06**	**1.437**	**1.390**	**0.654**					**+**			**+**	**+**		
**VRE11**	**0.043**	**0.477**	**+**					**+**	**+**	**+**				
**VRE13**		**0.014**	**+**							**+**				
**VRE19**		**1.404**	**+**							**+**		**+**		
**VRE20**	**1.284**	**1.142**	**0.835**					**+**			**+**	**+**		
**VRE24**	**+**	**0.620**	**+**					**+**		**+**	**+**	**+**		
**VRE25**		**+**	**+**								**+**	**+**	**+**	
**VRE29**	**1.361**	**0.587**	**+**											
**VRE30**	**0.965**	**+**	**0.378**										**+**	
**# of Strains Infected**	**11/21**	**16/21**	**9/21**	**1/21**	**1/21**	**1/21**	**1/21**	**3/21**	**1/21**	**3/21**	**2/21**	**2/21**	**1/21**	**2/21**

All VRE (vancomycin resistant) were obtained from the CDC & FDA Antimicrobial Resistance Isolate Bank. The remainder of the bacteria were obtained from ATCC. Strains 4082 and OG1RF are orally derived, 19434, 35567, and 49624 are *E. faecium*. A wide range of strains with various genetic differences are included to highlight the broad applicability of these phage, such as VRE strains which are common in hospital settings and pose a significant health risk but are not commonly associated with endodontic infections. Testing a wide range also highlights which phage are more useful in targeted versus broad spectrum treatments. A (+) indicates lysis of the bacteria was observed to any degree during spot testing. A blank cell indicates no lysis was observed during spot testing and no further testing was done, including EoP. Subsequent efficiency of plating (EoP) was calculated as EoP = PFU against test strain/PFU against reference strain. Reference strains for each phage are italicized. By definition EoP against reference strains is equivalent to 1.

### Growth curves

Five phages Pumpjack, GiddyUp, Revolver, AllMyExes, and Riverwalk were tested for their ability to inhibit the growth of OG1RF over a 21-hour period based on host range testing. The bacteria-only control yielded an average of 2.86 × 10^9^ CFU/ml, whereas the ampicillin control produced 3.97 × 10^6^ CFU/ml, representing a 99.9% reduction (S1 Table). All significance comparisons in S1 Table were made against the bacteria-only control.

**Fig 2:**
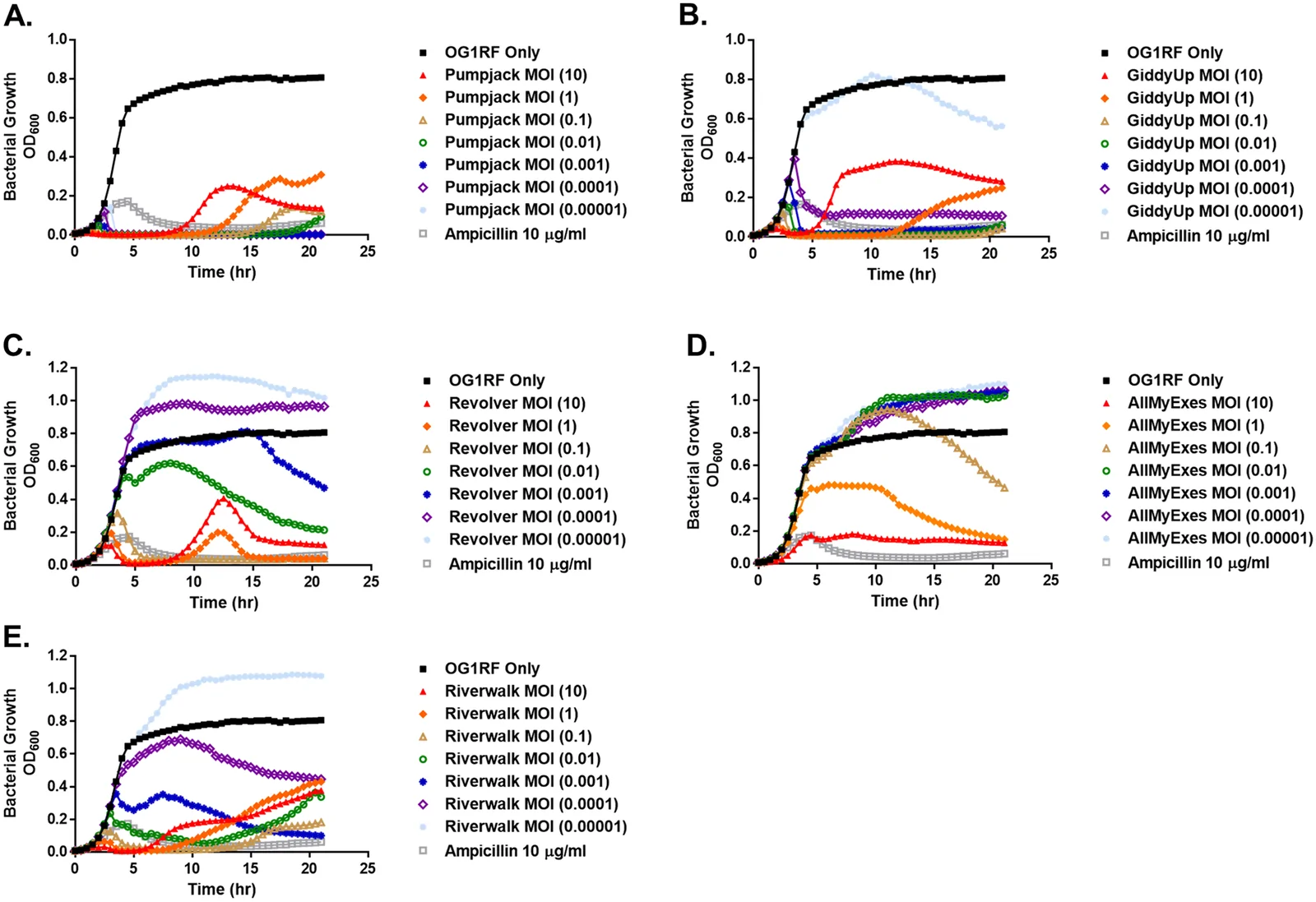
Phage mediated growth curves of OG1RF. A total of 1.1 × 10^6^ CFUs of bacteria were mixed with several amounts of phage ranging from 1.1 × 10^7^ PFU to 11 PFU making multiplicities of infection (MOI) 10-0.00001. OD_600_ was measured over 21 hr. Five phage were tested as follows **(A)** Pumpjack (n = 3), **(B)** GiddyUp (n = 3), **(C)** Revolver (n = 3), **(D)** AllMyExes (n = 3), and **(E)** Riverwalk (n = 3). The same bacteria only (n = 11) and ampicillin (n = 11) controls are shown in all five graphs.

**Fig 3:**
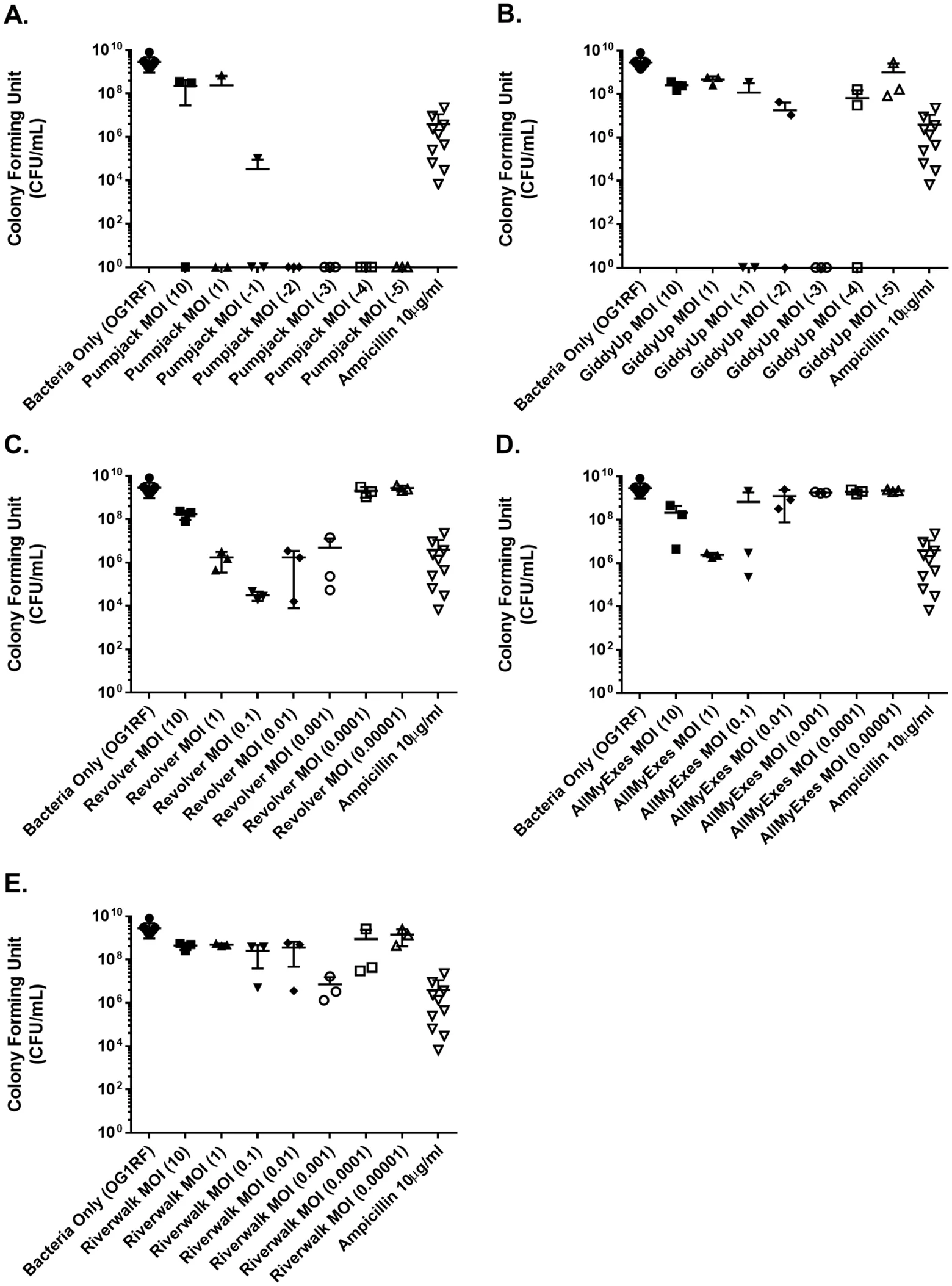
Individual data points of colony forming unit counts of phage mediated inhibition of OG1RF. A total of 1.1 × 10^6^ CFUs of bacteria were mixed with several amounts of phage ranging from 1.1 × 10^7^ PFU to 11 PFU making multiplicities of infection (MOI) 10-0.00001. After 21 hours of growth curve testing, samples were serially diluted, plated, and colonies counted to determine bacterial survival. Phages **(A)** Pumpjack, **(B)** GiddyUp, **(C)** Revolver, **(D)** AllMyExes, and **(E)** Riverwalk were all tested (n = 3). Bacteria only and ampicillin controls had n = 10. Data are shown as individual replicates and mean ± SD.

Pumpjack (Figs 2A and 3A) and GiddyUp (Figs 2B and 3B) eliminated all observable OG1RF at exceptionally low MOIs (0.01–0.00001). Revolver (Figs 2C and 3C) was effective at MOIs of 0.001–1 (> 99.8% reduction, 3.13 × 10^4^–4.87 × 10^6^ CFU/ml) but ineffective at lower MOIs. AllMyExes (Figs 2D and 3D) was most effective at an MOI of 1 (99.9% reduction, 2.40 × 10^6^ CFU/ml), while only moderate or non-significant reductions were observed at lower MOIs. Riverwalk (Figs 2E and 3E) was most effective at an MOI of 0.001 (99.8% reduction, 7.10 × 10^6^ CFU/ml), but both higher and lower MOIs showed reduced effectiveness. For reference, all titers (CFU/ml) and percent changes can be found in S1 Table.

Eight phages AllMyExes, Riverwalk, Revolver, PricklyPear, TexasRanger, Haystack, Stockyards, and RioGrande were tested against strain 4082. Despite not producing plaques during efficiency of plating experiments, TexasRanger was included due to inhibitory activity seen during spot testing. The bacteria-only control produced 2.81 × 10^9^ CFU/ml, while the ampicillin control yielded 9.16 × 10^6^ CFU/ml (99.7% reduction) (S2 Table).

**Fig 4:**
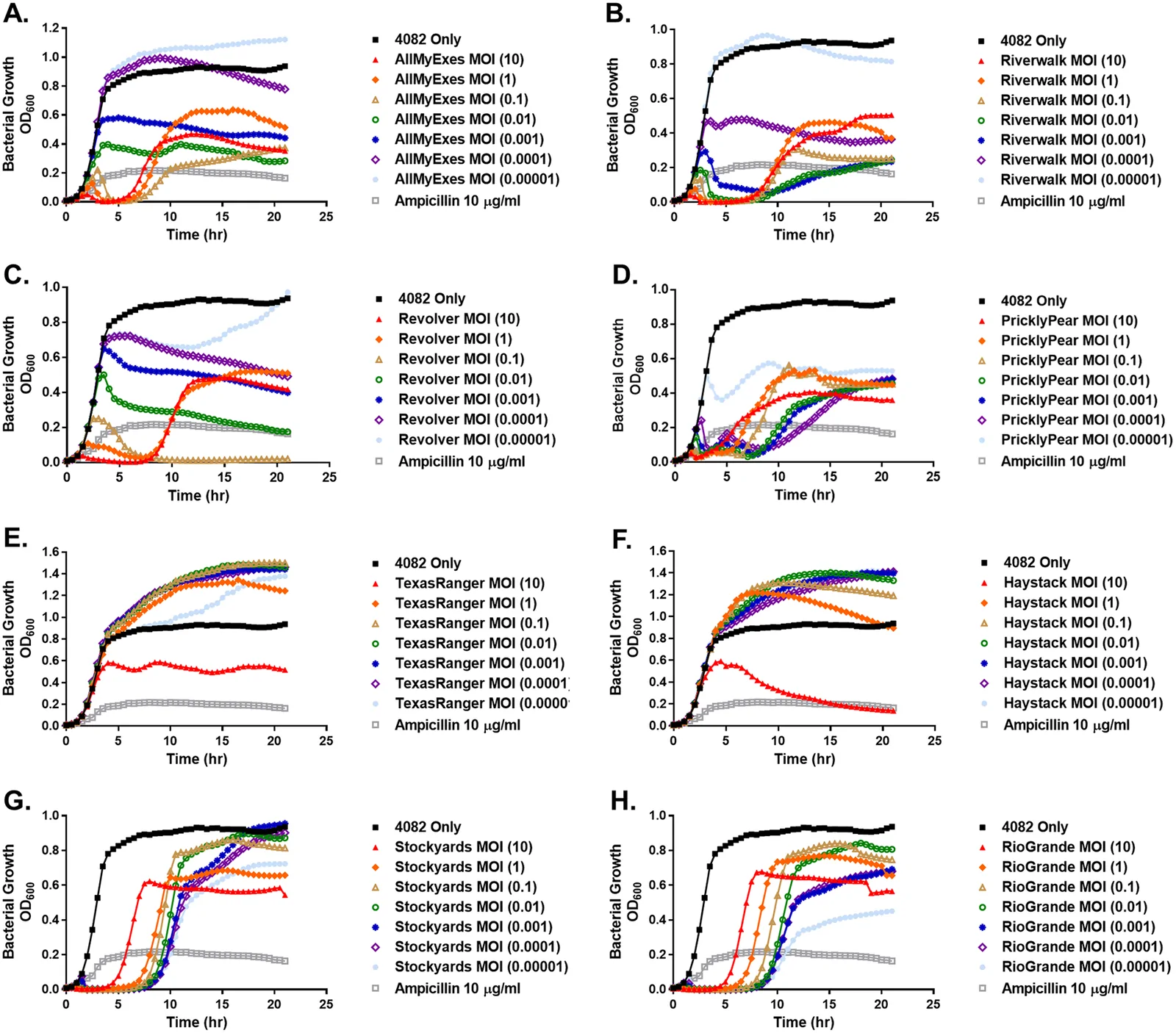
Phage mediated growth curves of 4082. A total of 1.1 × 10^6^ CFUs of bacteria were mixed with several amounts of phage ranging from 1.1 × 10^7^ PFU to 11 PFU making multiplicities of infection (MOI) 10-0.00001. OD_600_ was measured over 21 hr. Eight phage were tested as follows **(A)** AllMyExes, **(B)** Riverwalk, **(C)** Revolver, **(D)** PricklyPear, and **(E)** TexasRanger, **(F)** Haystack, **(G)** Stockyards, and **(H)** RioGrande, n = 3 for all phage. The same bacteria only (n = 9) and ampicillin (n = 9) controls are shown in all eight graphs.

**Fig 5:**
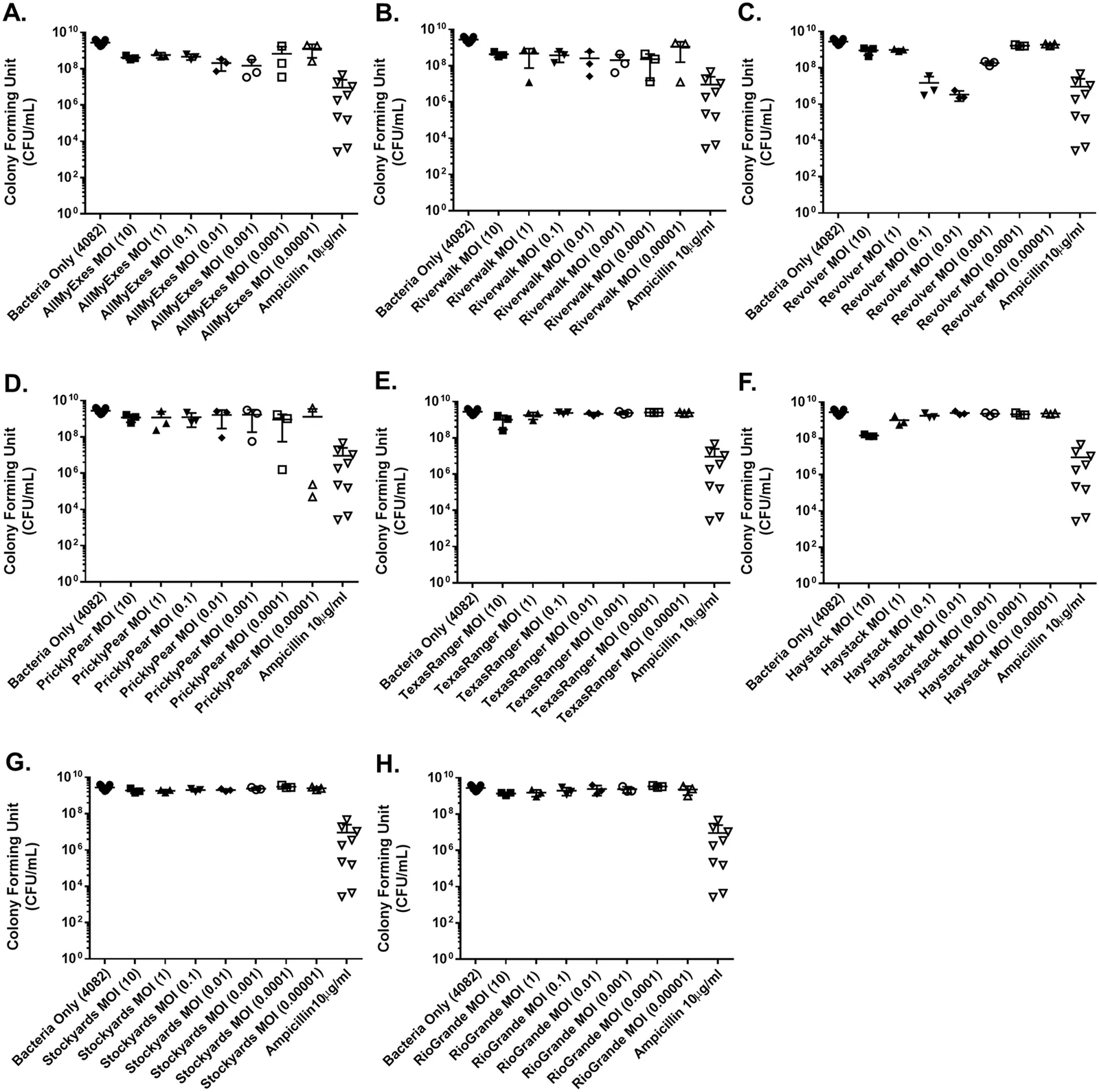
Individual data points of colony forming unit counts of phage mediated inhibition of 4082. A total of 1.1 × 10^6^ CFUs of bacteria were mixed with several amounts of phage ranging from 1.1 × 10^7^ PFU to 11 PFU making multiplicities of infection (MOI) 10-0.00001. After 21 hours of growth curve testing, samples were serially diluted, plated, and colonies counted to determine bacterial survival. Phages **(A)** AllMyExes, **(B)** Riverwalk, **(C)** Revolver, **(D)** PricklyPear, **(E)** TexasRanger, **(F)** Haystack, **(G)** Stockyards, and **(H)** RioGrande were all tested (n = 3). Bacteria only and ampicillin controls had n = 9. Data are shown as individual replicates and mean ± SD.

AllMyExes (Figs 4A and 5A) and Riverwalk (Figs 4B and 5B) showed statistically significant decrease in CFU counts at all MOIs but achieved maximum inhibition of 93% and 95% (2.05 × 10^8^ CFU/ml, 1.48 × 10^8^ CFU/ml) at an MOI of 0.001. According to CFU counts, Revolver (Figs 4C and 5C) performed best at an MOI of 0.01, with a 99.9% reduction (3.41 × 10^6^ CFU/ml). PricklyPear (Figs 4D and 5D) was most effective at an MOI of 0.0001, with an approximate 68% reduction (9.11 × 10^8^ CFU/ml). TexasRanger (Figs 4E and 5E) and Haystack (Figs 4F and 5F) both showed their greatest inhibition at an MOI of 10, achieving 64% and 95% reductions (1.02 × 10^9^ CFU/ml, 1.45 × 10^8^ CFU/ml, respectively). Both Stockyards (Figs 4G and 5G) and RioGrande (Figs 4H and 5H) showed no statistically significant decreases in CFU counts, though reductions ranged from approximately 11–51%. For both phages, at an MOI of 0.0001, an increase in bacterial counts was observed. Despite no significant differences for these two phages, OD readings at all MOIs were approximately less than or equal to those of the bacteria-only control. For reference, all titers (CFU/ml) and percent changes can be found in S2 Table. Additionally, all OD and individual replicate titer counts for all experiments can be found in S3 File.

### Biofilm disruption

**Fig 6:**
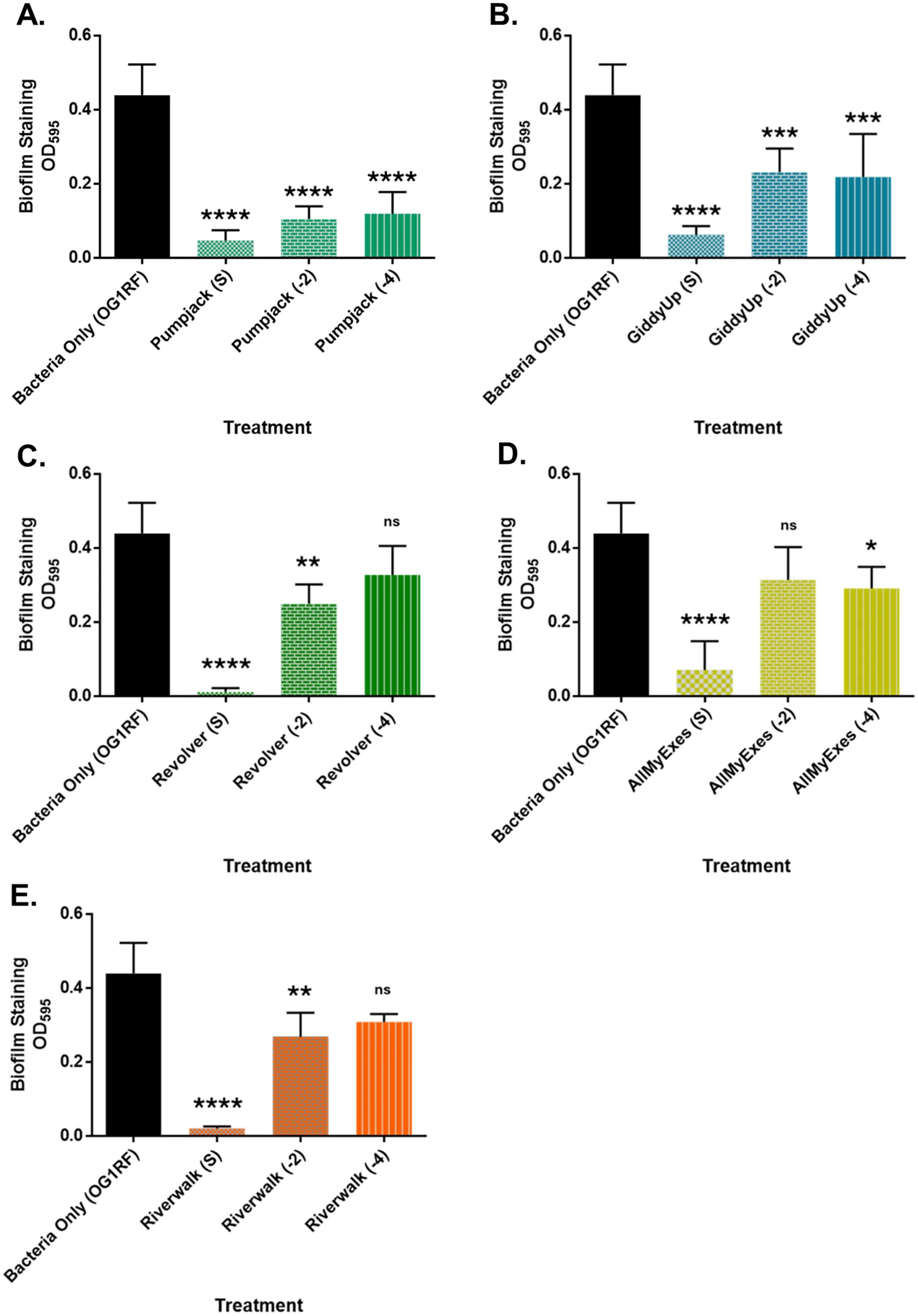
Disruption of OG1RF produced biofilm. Mature biofilms were grown over two weeks with an initial inoculation of 1.1 × 10^6^ CFU of bacteria. Phage were added as (S) 10^7^ PFU, (−2) 10^5^ PFU, or (−4) 10^3^ PFU and incubated for one week before crystal violet staining and measuring OD_595_. Technical triplicate was used for all testing. An n = 3 was used for all phage treatments, **(A)** Pumpjack, **(B)** GiddyUp, **(C)** Revolver, **(D)** AllMyExes, and **(E)** Riverwalk. The bacteria only control had n = 7. Data were analyzed with a one-way ANOVA and Dunnett’s post-hoc test (bacteria only comparison), ns = not significant, (*) p ≤ 0.05, (**) p ≤ 0.01, (***) p ≤ 0.001, (****) p ≤ 0.00001. Error bars represent standard deviation.

The same phage–bacteria pairs used in growth curves and CFU counts were also used to test phage–mediated disruption of mature biofilms. Generally, all phages against either host showed decreased effectiveness as phage doses applied to biofilms decreased from 10^7^ PFU to 10^5^ or 10^3^ PFU. The following percentage decreases were observed compared to OG1RF only: Pumpjack 73–89% (Fig 6A), GiddyUp 47–86% (Fig 6B), Revolver 26–97% (Fig 6C), AllMyExes 29–84% (Fig 6D), and Riverwalk 30–96% (Fig 6E).

**Fig 7:**
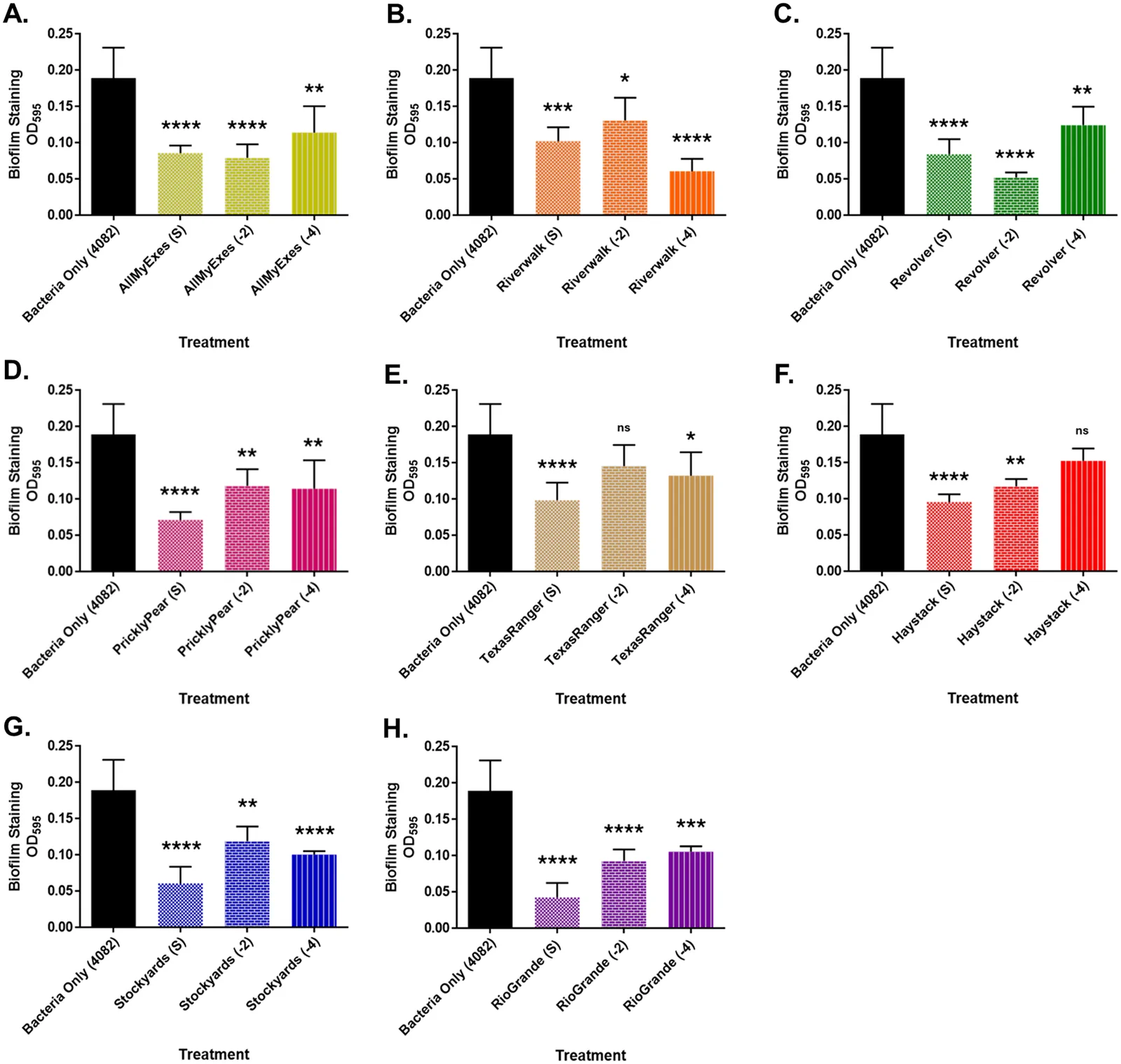
Disruption of 4082 Produced Biofilm. Mature biofilms were grown over two weeks with an initial inoculation of 1.1 × 10^6^ CFU of bacteria. Phage were added as (S) 10^7^ PFU, (−2) 10^5^ PFU, or (−4) 10^3^ PFU and incubated for one week before crystal violet staining and measuring OD_595_. Technical triplicate was used for all testing. An n = 3 was used for all phage treatments, **(A)** AllMyExes, **(B)** Riverwalk, **(C)** Revolver, **(D)** PricklyPear, **(E)** TexasRanger, **(F)** Haystack, **(G)** Stockyards, **(H)** RioGrande. The bacteria only control had n = 12. Data were analyzed with a one-way ANOVA and Dunnett’s post-hoc test (bacteria only comparison), ns = not significant, (*) p ≤ 0.05, (**) p ≤ 0.01, (***) p ≤ 0.001, (****) p ≤ 0.00001. Error bars represent standard deviation.

For strain 4082, biofilm reductions were measured as follows compared to bacteria-only control: AllMyExes 40–58% (Fig 7A), Riverwalk 31–68% (Fig 7B), Revolver 34–72% (Fig 7C), PricklyPear 37–62% (Fig 7D), TexasRanger 23–48% (Fig 7E), Haystack 19–49% (Fig 7F), Stockyards 37–68% (Fig 7G), and RioGrande 44–77% (Fig 7H). Two different phage loads did not show statistically significant differences compared to bacteria-only control: Haystack 10^3^ PFU (19%) and TexasRanger 10^5^ PFU (23%). All significance comparisons shown in Figs 6 and 7 were made against the bacteria-only controls. Individual replicate OD measurements can be found in S4 File.

## Discussion

This study characterized 14 *E. faecalis* phages using transmission electron microscopy, host range analysis, bacterial growth inhibition, and biofilm disruption assays. *E. faecalis* is an important human pathogen associated with diverse infections, including endodontic infections that are often overlooked. Several of the phages tested here effectively prevented bacterial growth and disrupted preformed biofilms of two orally derived *E. faecalis* strains, one of which was isolated from a root canal.

Compared with phages reported in recent years, those described here performed favorably across several metrics. The phages in this study displayed variable host ranges, infecting between one and 16 strains. Revolver, AllMyExes, and Riverwalk were particularly effective, infecting 9, 11, and 16 strains, respectively, in efficiency of plating experiments. Recently reported phages EF1TV [38], EfKS5 [39], and ZEFP [40] all had relatively large host ranges infecting upwards of 21 strains. Based on host range, ours and these phages would be considered the best candidates to have on hand in a treatment facility to cover the variety of clinical infections. The phage in the *Efquatrovirus* and *Saphexavirus* genera, which have narrow host ranges may still be helpful when used in a cocktail of phages or screened against a patient’s infectious strain after it is isolated. This appears particularly true for the saphexaviruses Pumpjack and GiddyUp, which were able to completely eliminate any observable OG1RF, an orally derived strain, at low multiplicity of infections (MOIs) (e.g., 0.00001), potentially giving them a specialist role in a treatment regimen. Revolver also performed well, reducing OG1RF to 3.13 × 10⁴ CFU/ml at an MOI of 0.1. Biofilm disruption against OG1RF was consistently high, ranging from 84–97% among five phages tested at 10⁷ PFU.

Against strain 4082, isolated from a root canal, Riverwalk, AllMyExes, and Revolver significantly reduced bacterial loads in growth curve experiments even at low MOIs, with Revolver showing a 99.9% reduction at an MOI of 0.01. Top biofilm disruption rates across the eight phages tested ranged from 48–77%. The study concerning phage EfKS5 also performed growth inhibition assays but observed elimination only at MOIs ≥ 1, and only going as low as 0.001 compared to 0.00001 in our work. Phages EF1TV and ZEFP, in addition to EfKS5 also showed biofilm reduction efficacy approximately 50–68%, decidedly lower than 77–97% in this study.

When looking at bacterial inhibition it is important to note the variety of MOI dependent trends observed amongst the different phage. For example, against OG1RF we can see Revolver and AllMyExes, and against 4082 we see Haystack provide the classic pattern of efficacy at high MOI and dropping off at low MOI. However, several other phage elicit various unique patterns. Against OG1RF, the phage Riverwalk appears to perform optimally at MOI 0.001 with a drop off in efficacy at both higher and lower MOIs. Pumpjack, while effective at high MOIs, appears to perform better at an MOI of 0.01 or lower. Against 4082, PricklyPear is the least effective at MOIs 0.001 and 0.01 but then nominally improves at higher and lower MOIs. Revolver, much like Riverwalk mentioned above, has the inverse pattern and is most effective at MOI 0.01 but then drops off in efficacy at higher and lower MOIs. These trends highlight the complicated phage-host relationship that have a critical impact on phage therapy and emphasize the need to characterize phage to determine potentially optimal dosages when moving into *in vivo* experiments. There are two mechanisms that may be playing a role in the trends seen in this work, namely, lysis inhibition and lysis from without. Lysis inhibition is a mechanism where secondary adsorption of phage onto an already-infected host cell at high phage-to-bacterium ratios delays the onset of lysis. While this can increase the eventual phage burst size, the extended latent period can result in a higher bacterial count at a fixed endpoint. Lysis from without is the rapid depletion of a bacterial population, but not necessarily complete eradication, caused by a high phage-to-bacterium ratio and an abundance of bacteria killing lysins. This rapid depletion through lysins does not allow for high levels of initial phage infection and amplification [44]. The potential role that these mechanisms play in this work is not explored but serves as a reminder for consideration in phage pharmacodynamics and pharmacokinetics in living systems.

Overall, our phages were comparable to previously reported phages in host range and bacterial inhibition, with several notable examples functioning at low MOIs. Importantly, our phages generally achieved stronger biofilm reduction even at low concentrations. It is important to note that biofilm disruption was quantified only with crystal violet staining, which only measures total biomass and cannot differentiate between live and dead cells. Future experiments in more biologically relevant models, accounting for more variables, such as *ex vivo* teeth and potential animal work will incorporate live/dead staining and CFU counts. In relation to biologically relevant variables, a clearer understanding of phage efficacy in saliva or other oral suspensions will be critical. This work was performed in tryptic soy broth which differs greatly in composition from saliva and endodontic spaces, which may result in deactivated phage compared to lab formulated media. A better understanding in this area will aid the transition from benchtop to clinical application.

As with all phage characterization studies, a key limitation is the restricted number of strains tested. Here, strains 4082 and OG1RF were emphasized due to their oral origin, making them particularly relevant to endodontic infections. The variability in host range, from the broad activity of Revolver to the narrow specificity of the *Efquatrovirus* phages, likely reflects differences in the cell surface receptors that these phages use for adsorption, a key determinant of a phage’s lytic cycle. Expanding testing to larger panels of clinical isolates will strengthen future work, though it will remain limited until clinical results can be observed. *In vitro* assays also face limitations because phages have a short half-life *in vivo* due to host clearance and deactivation, which does not occur *in vitro*.

While the *in vitro* data here demonstrate potential, translation into clinical practice raises distinct challenges related to delivery and stability. Intravenous delivery for systemic infections is relatively straightforward, though questions remain regarding phage pharmacokinetics, pharmacodynamics, immune responses, and the unique capacity of phages to replicate [45]. For RCT, the major questions are when and how should phage be applied to a root canal? The simplest approach is irrigating canals with phages prior to sealing. This would minimize additional time and workload added to the clinician and patient as irrigation is already an integral step to the procedure. In the investigation concerning phage ZEFP, it was shown that a phage irrigant alone was more effective at killing *E. faecalis* than phage + sodium hypochlorite or sodium hypochlorite + EDTA (another irrigant) [40]. Alternatively, to protect the phage and potentially allow for extended efficacy phage could be suspended in biocompatible materials and placed in canals. One study incorporated phages into a thermoreversible hydrogel for insertion into canals that require multiple visits, achieving sustained release and antibacterial/antibiofilm activity in a rat RCT model [46]. Another study utilized chitosan-alginate microspheres that stabilized phage for up to 6 hr in artificial saliva in addition to improved stability in pH levels from 2-7 and temperatures 4–60 °C [47].

Beyond standard phage characterization the intersection of phage therapy and clinical endodontic practice is new. As such future studies should test phage stability, compatibility, antibacterial/antibiofilm activity in the presence of endodontic sealers. Key unanswered questions include whether phages remain effective in contact with sealers, whether incorporation affects sealer mechanical properties, and whether phages can be embedded into sealers for long-term activity. Phage pH dependent stability, not tested in this investigation, will be tested in conjunction with sealers as it will be more clinically relevant to this application than pH testing alone. Furthermore, the bacterial regrowth observed in the OD_600_ growth curves following initial phage-mediated lysis is a classic indicator of the selection for and outgrowth of phage-resistant bacterial mutants. While this study did not characterize the genetic basis of resistance, this observation underscores the critical need for using phage cocktails in a clinical setting to overcome the inevitable evolution of bacterial resistance. Overall, we intend to move beyond the characterization stage and test phage at various points in the RCT process including irrigation alone and in conjunction with common irrigants mentioned previously, biomaterial injection, and incorporation into endodontic sealers such as AH Plus Jet, Endosequence BC, Pulp Canal, and GuttaFlow in *ex vivo* teeth models and small animal models. We will test both mono-phage treatment and phage cocktails to address synergy between the phage. Phage cocktails utilize two or more phage and are generally considered superior to single phage treatments.

Overall, phage characterization, as performed here and in other studies, represents an essential first step toward clinical translation. Based on their broad host ranges, biofilm reduction capacities, and absence of temperate lifestyle, virulence, or antimicrobial resistance genes, Revolver, Riverwalk, and AllMyExes show therapeutic potential. Ultimately, continued work addressing the outstanding questions above will be critical for advancing phage therapy in RCT and beyond.

## Materials and methods

### Bacteria, phages, and isolation conditions

Environmental phage enrichment cultures were prepared by incubating 1 ml of unfiltered wastewater with 1 ml of the following ATCC *E. faecalis* strains for 24 hr at 37 °C in 10 ml of Tryptic Soy Broth (TSB): 33186, 29212, 19433, or 47077/OG1RF (Table 3). Cultures were subsequently centrifuged at 4800 × g, the lysate collected, serially diluted 10-fold four times, and each dilution plated with its respective host (200 µl overnight culture + 10 µl phage dilution) using 4 ml of diluted Tryptic Soy Agar (4%) in a double agar overlay method with a TSA base. Plaques were picked, amplified, and plated three times to ensure isolation of a single phage species (Table 4). High-titer lysates were produced by inoculating 10 ml of TSB with 500 µl of overnight bacterial culture and initially picking a plaque or adding 100 µl phage stock for subsequent amplifications. Titers were performed to verify a minimum concentration of 10^8^ PFU/ml.

**Table 3:** Bacterial strains utilized.

Strain	Species	Source	Biosample Accession #	Known Resistance Mechanisms
4082	*E. faecalis*	ATCC		
47077/OG1RF	*E. faecalis*	ATCC		
29212	*E. faecalis*	ATCC		
33186	*E. faecalis*	ATCC		
19433	*E. faecalis*	ATCC		
51299	*E. faecalis*	ATCC		
49624	*E. faecium*	ATCC		
35667	*E. faecium*	ATCC		
19434	*E. faecium*	ATCC		
VRE01	*E. faecalis*	CDC	SAMN15040080	tet(L), tet(M), VanA
VRE02	*E. faecalis*	CDC	SAMN15040081	aac(6’)-le, ant(6)-la, aph(3’)-IIIa, catA8, qacZ, VanB
VRE05	*E. faecalis*	CDC	SAMN15040084	aac(6’)-le, ant(6)-la, aph(3’)-IIIa, catA7, erm(B), lsa(A), mph(D), sat4, tet(M), Z
VRE06	*E. faecalis*	CDC	SAMN15040085	aac(6’)-le, erm(B), lsa(A), mph(D), qacZ, VanB
VRE11	*E. faecalis*	CDC	SAMN15040090	tet(M), VanB
VRE13	*E. faecalis*	CDC	SAMN15040092	---
VRE19	*E. faecalis*	CDC	SAMN15040098	aac(6’)-le, ant(6)-la, aph(3’)-IIIa, catA7, sat4, tet(L), tet(M), VanB, Z
VRE20	*E. faecalis*	CDC	SAMN15040099	aac(6’)-le, qacZ, VanB
VRE24	*E. faecalis*	CDC	SAMN15040103	aac(6’)-le, ant(6)-la, aph(3’)-IIIa, catA7, sat4, tet(L), tet(M), VanB, Z
VRE25	*E. faecalis*	CDC	SAMN15040104	---
VRE29	*E. faecalis*	CDC	SAMN15040108	aac(6’)-le, ant(4’)-la, ant(6)-la, aph(3’)-IIIa, VanA
VRE30	*E. faecalis*	CDC	SAMN15040109	dfrF, STR, tet(L), tet(M)

The CDC strains are all part of the Vancomycin-Resistant Enterococci panel (CDC & FDA AR Isolate Bank) [48].

**Table 4:** Phage species utilized.

Name	Isolation Host	Point of Isolation	GenBank Accession #
Alamo	ATCC 29212	USA: Walnut Creek Wastewater Treatment Plant, Austin, TX GPS: 30.27916, −97.64922	PV544170
Haystack	ATCC 33186	USA: Village Creek Water Reclamation Facility, Arlington, TX GPS: 32.77303, −97.14422	PV544173
LoneStar	ATCC 29212	USA: South Austin Regional Wastewater Treatment Plant, Del Valle, TX GPS: 30.20980, −97.60828	PV544174
PricklyPear	ATCC 33186	USA: South Austin Regional Wastewater Treatment Plant, Del Valle, TX GPS: 30.20980, −97.60828	PV544175
RioGrande	ATCC 19433	USA: Unitec Wastewater Treatment Plant, Laredo, TX GPS: 27.70213, −99.44805	PV544178
Stockyards	ATCC 19433	USA: Village Creek Water Reclamation Facility, Arlington, TX GPS: 32.77303, −97.14422	PV544180
TexasRanger	ATCC 33186	USA: Village Creek Water Reclamation Facility, Arlington, TX GPS: 32.77303, −97.14422	PV544181
TwoStep	ATCC 29212	USA: Zacate Creek Wastewater Treatment Facility, Laredo, TX GPS: 27.49984, −99.49286	PV544182
Vaquero	ATCC 33186	USA: Zacate Creek Wastewater Treatment Facility, Laredo, TX GPS: 27.49984, −99.49286	PV544183
GiddyUp	ATCC 29212	USA: Unitec Wastewater Treatment Plant, Laredo, TX GPS: 27.70213, −99.44805	PV544172
Pumpjack	ATCC 47077/OG1RF	USA: Walnut Creek Wastewater Treatment Plant, Austin, TX GPS: 30.27916, −97.64922	PV544176
AllMyExes	ATCC 33186	USA: North Laredo Wastewater Plant, Laredo, TX GPS: 27.59653, −99.46260	PV544171
Riverwalk	ATCC 29212	USA: Laredo South Side Wastewater, Laredo, TX GPS: 27.44630, −99.48943	PV544179
Revolver	ATCC 33186	USA: Zacate Creek Wastewater Treatment Facility, Laredo, TX GPS: 27.49984, −99.49286	PV544177

In addition to the four previously mentioned bacterial strains, 17 other *E. faecalis* and *E. faecium* strains obtained from ATCC and the CDC were used in various experiments (Table 3). The CDC strains are all part of the Vancomycin-Resistant Enterococci panel (CDC & FDA AR Isolate Bank). All genomic characterization, accession numbers, and resistance profiles are available on the AR Isolate Bank website (https://wwwn.cdc.gov/ARIsolateBank/) while accession numbers and resistance mechanisms can be found in Table 3. Isolates were identified by the CDC using Matrix-Assisted Laser Desorption Ionization-Time of Flight Mass Spectrometry (MALDI-TOF MS) [48]. A wide range of strains with various genetic differences are included to highlight the broad applicability of these phage, such as VRE strains which are common in hospital settings and pose a significant health risk but are not commonly associated with endodontic infections. Testing a wide range also highlights which phage are more useful in targeted versus broad spectrum treatments.

### Lysogenic, virulent, and antimicrobial resistance gene detection

Phage genomes were manually inspected for genes that could confer a lysogenic lifestyle to the phage, toxins to the host, or antimicrobial resistance. Two online programs, PhageAI [42] and PhageLeads [43] were also used to analyze phage genomes.

### Transmission electron microscopy

Phage stocks were freshly prepared, titered, and sent for TEM imaging at the Keith R. Porter Imaging Facility, University of Maryland, Baltimore County. A total of 10 µl of lysate was placed onto 200-mesh formvar-covered, carbon-coated copper grids (EMS, Hatfield, PA, USA), incubated for 1 min, briefly rinsed with ultrapure water, and stained with 2% uranyl acetate for 2 min.

Samples were imaged on a Hitachi HT7800 120 kV TEM equipped with an AMT NanoSprint15B digital camera. ImageJ (v1.54g) was used to measure the length and width of the capsid head and tail. A minimum of three images was used for all measurements, except for RioGrande with n = 2.

### Host range and efficiency of plating

Each bacterial strain from Table 3 was grown overnight at 37 °C with shaking, and then 200 µl of culture was plated in 4 ml of molten TSA. After solidification, 5 µl of phage stock normalized to 10^8^ PFU/ml was pipetted onto the agar surface, allowed to dry, and then incubated for 24 hr at 37 °C. Three independently prepared stocks for each phage were used for spotting (n = 3). Efficiency of plating was performed by mixing 10 µl of phage at 10^8^-10^4^ PFU/ml with 200 µl of bacteria, incubated for 30 min, plated in 4 ml of molten TSA, and incubated for 24 hr at 37 °C. Plaques were counted and efficiency of plating was calculated as the ratio of “PFU against test strain/PFU against reference strain”.

### Growth curves

Phage-mediated inhibition of *E. faecalis* strains OG1RF, which was orally derived [49], and 4082, which was isolated from a root canal (https://www.atcc.org/products/4082), was measured through optical density (OD_600_) and colony forming unit (CFU) counts. Bacterial cultures were grown overnight in TSB, diluted 100-fold in 10 ml of TSB, and grown to an OD_600_ of 0.1 as read by a BioTek Synergy H1 (Agilent, Santa Clara, CA, USA) plate reader with 100 µl of background-corrected culture. An OD_600_ of 0.1 was determined to be equivalent to 1.1 × 10^8^ CFU/ml during optimization steps. Phages were normalized to 10^8^ PFU/ml and serially diluted 10-fold to 10^2^ PFU/ml. In 96-well plates (Cat# 266120, Thermo Fisher Scientific, Waltham, MA, USA) 10 µl (1.1 × 10^6^ CFU) of bacteria and 110 µl (1.1 × 10^7^ to 11 PFU) of each phage concentration were mixed and brought to a total volume of 200 µl with TSB. The multiplicity of infection of these combinations ranged from 10 to 0.00001. Bacteria-only and ampicillin controls (10 µg/ml) were included in addition to blank correction wells, on each test plate. OD_600_ was measured in a BioTek Synergy H1 plate reader every 30 min for 21 hr with constant shaking at 37 °C. All controls and phage-treated samples were performed in technical triplicate. Three independently prepared stocks, biological replicates, for each phage were tested on separate plates, n = 3. Bacteria-only and ampicillin controls were included on each plate to account for inter-plate variability. A total of nine plates were needed to test phage against strain 4082 and eleven plates to test against strain OG1RF, this resulted in n = 9 (4082) and n = 11 (OG1RF) for both controls. Data were analyzed by first averaging technical replicates and then averaging biological replicates. To quantify viable bacteria, at the end of the 21-hr time course, samples were taken from the wells of each control and test condition. Specifically, for each biological replicate (n = 3), a sample was taken from the technical replicate well that exhibited the median OD_600_ reading. The next day colonies were counted. The number of biological replicates for CFU counts of bacteria treated with phage matched the growth curves (n = 3). The OG1RF bacterial control and associated ampicillin control had n = 10 compared with n = 11, which was used in growth curves. This change in replicate number was due to agar plate damage before counting could occur.

### Biofilm disruption assays

Overnight cultures of OG1RF and 4082 were prepared as described in Growth Curves, and 10 µl of normalized culture was diluted into 990 µl of TSB in wells of a 24–well tissue-culture-treated plate (Cat# 353047) (Corning, Corning, NY, USA). Plates were incubated statically at 37 °C with 5% CO_2,_ for two weeks to establish mature biofilms. After two weeks the medium was replaced with fresh TSB and in triplicate infectious phages based on host range were added at 10^7^, 10^5^, or 10^3^ PFU and incubated for one week. A bacteria–only control was also included. In all cases bacterial growth was observed after one week. Biofilm disruption was characterized by crystal violet staining as follows: Medium was removed, and wells were washed three times with 1 ml of sterile water. Then, 1 ml of 0.25% crystal violet in methanol was added to each well and incubated for 30 min. Next, crystal violet was removed, and wells were washed with 1 ml of water four times and allowed to dry. Finally, 1 ml of 30% acetic acid was added to each well to solubilize the crystal violet, and OD_595_ was measured. All samples were tested in technical triplicate and all phage-bacteria combinations were carried out in biological triplicate (n = 3). The OG1RF–only control had n = 7, and the 4082–only control had n = 12.

### Statistical analysis

All statistical analyses was performed using GraphPad Prism v6.07. CFU data were analyzed with a one-way ANOVA and Dunnett’s post-hoc test (bacteria only comparison) for each strain and all associated phages at all MOIs. Biofilm data were analyzed with a one-way ANOVA and Dunnett’s post-hoc test (bacteria-only comparison) for each strain-phage combination. A p value ≤ 0.05 was considered statistically significant. Standard deviation was used to show variance in all experiments, tables, and graphs.

## Supporting information

S1 Fig Transmission electron microscopy images of 11 remaining phage. (A) AllMyExes, (B) Riverwalk, (C) LoneStar, (D) TwoStep, (E) Vaquero, (F) PricklyPear, (G) TexasRanger, (H) Haystack, (I) RioGrande, (J) Stockyards, (K) Pumpjack. Imaging was performed at the Keith R. Porter Imaging Facility at the University of Maryland, Baltimore County by Dr. Tagide deCarvalho.
(TIF)

S1 Table Colony forming unit counts of phage mediated inhibition of OG1RF. A total of 1.1 × 10^6^ CFUs of bacteria were mixed with several amounts of phage ranging from 1.1 × 10^7^ PFU to 11 PFU making multiplicities of infection (MOI) 10-0.00001. After 21 hours of growth curve testing, samples were serially diluted, plated, and colonies counted to determine bacterial survival. Phages Pumpjack, GiddyUp, Revolver, AllMyExes, and Riverwalk were all tested (n = 3). Bacteria only and ampicillin controls had n = 10. Data were analyzed with a one-way ANOVA and Dunnett’s post-hoc test (bacteria only comparison), ns = not significant, (*) p ≤ 0.05, (**) p ≤ 0.01, (***) p ≤ 0.001, (****) p ≤ 0.00001. Data are shown as mean ± SD.
(PDF)

S2 Table Colony forming unit counts of phage mediated inhibition of 4082. A total of 1.1 × 10^6^ CFUs of bacteria were mixed with several amounts of phage ranging from 1.1 × 10^7^ PFU to 11 PFU making multiplicities of infection (MOI) 10-0.00001. After 21 hours of growth curve testing, samples were serially diluted, plated, and colonies counted to determine bacterial survival. Phages AllMyExes, Riverwalk, Revolver, PricklyPear, TexasRanger, Haystack, Stockyards, and RioGrande were all tested (n = 3). Bacteria only and ampicillin controls had n = 9. Data were analyzed with a one-way ANOVA and Dunnett’s post-hoc test (bacteria only comparison), ns = not significant, (*) p ≤ 0.05, (**) p ≤ 0.01, (***) p ≤ 0.001, (****) p ≤ 0.00001.
(PDF)

S1 File Phage morphology measurements. (XLSX)

S2 File Efficiency of plating calculations. (XLSX)

S3 File Growth curve ODs and CFU titers. (XLSX)

S4 File Biofilm disruption measurements. (XLSX)
