# Supplementary material for: Phage mediated growth inhibition and biofilm disruption of the endodontic pathogen Enterococcus faecalis
Source: PLoS One. 2026 Jun 17;21(6):e0350657. doi: 10.1371/journal.pone.0350657 (PMC13495677; doi:10.1371/journal.pone.0350657)
Supplement: Supplementary file 1 [file pone.0350657.s002.pdf]

S1 Table. Colony Forming Unit Counts of Phage Mediated Inhibition of OG1RF.

Treatment	CFU/ml	% Change vs. Bacteria	Significance
Bacteria Only (OG1RF)	2.86E+09 ± 1.93E+09	X	N/A
Pumpjack MOI (10)	2.25E+08 ± 1.97E+08	-92.1	****
Pumpjack MOI (1)	2.37E+08 ± 4.11E+08	-91.7	****
Pumpjack MOI (0.1)	3.33E+04 ± 5.77 E+04	-99.9	****
Pumpjack MOI (0.01)	Below Limit of Detection	----	****
Pumpjack MOI (0.001)	Below Limit of Detection	----	****
Pumpjack MOI (0.0001)	Below Limit of Detection	----	****
Pumpjack MOI (0.00001)	Below Limit of Detection	----	****
GiddyUp MOI (10)	2.58E+08 ± 1.17E+08	-91.0	***
GiddyUp MOI (1)	4.81E+08 ± 1.85E+08	-83.2	***
GiddyUp MOI (0.1)	1.18E+08 ± 2.04E+08	-95.9	****
GiddyUp MOI (0.01)	1.81E+07 ± 2.25E+07	-99.4	****
GiddyUp MOI (0.001)	Below Limit of Detection	----	****
GiddyUp MOI (0.0001)	6.46E+07 ± 8.66E+07	-97.7	****
GiddyUp MOI (0.00001)	1.02E+09 ± 1.55E+09	-64.4	*
Revolver MOI (10)	1.74E+08 ± 8.10E+07	-93.9	****
Revolver MOI (1)	1.73E+06 ± 1.38E+06	-99.9	****
Revolver MOI (0.1)	3.13E+04 ± 1.42E+04	-99.9	****
Revolver MOI (0.01)	1.72E+06 ± 1.71E+06	-99.9	****
Revolver MOI (0.001)	4.87E+06 ± 8.19E+06	-99.8	****
Revolver MOI (0.0001)	2.01E+09 ± 1.02E+09	-29.5	ns
Revolver MOI (0.00001)	2.72E+09 ± 7.77E+08	-4.8	ns
AllMyExes MOI (10)	2.08E+08 ± 2.25E+08	-92.7	****
AllMyExes MOI (1)	2.40E+06 ± 5.83E+05	-99.9	****
AllMyExes MOI (0.1)	6.68E+08 ± 1.15E+09	-76.6	**
AllMyExes MOI (0.01)	1.22E+09 ± 1.14E+09	-57.4	*
AllMyExes MOI (0.001)	1.78E+09 ± 1.16E+08	-37.7	ns
AllMyExes MOI (0.0001)	1.97E+09 ± 4.51E+08	-31.1	ns
AllMyExes MOI (0.00001)	2.18E+09 ± 2.50E+08	-23.8	ns
Riverwalk MOI (10)	4.40E+08 ± 1.59E+08	-84.6	***
Riverwalk MOI (1)	4.88E+08 ± 6.33E+07	-82.9	***
Riverwalk MOI (0.1)	2.55E+08 ± 2.17E+08	-91.1	***
Riverwalk MOI (0.01)	3.57E+08 ± 3.10E+08	-87.5	***

Riverwalk MOI (0.001)	7.10E+06 ±8.34E+06	-99.8	****
Riverwalk MOI (0.0001)	8.81E+08 ± 1.46E+09	-69.1	**
Riverwalk MOI (0.00001)	1.44E+09 ± 1.03E+09	-49.6	ns
Ampicillin 10 µg/ml	3.97E+06 ± 7.13E+06	-99.9	****

A total of 1.1×10^6 CFUs of bacteria were mixed with several amounts of phage ranging from 1.1×10^7 PFU to 11 PFU making multiplicities of infection (MOI) 10-0.00001. After 21 hours of growth curve testing, samples were serially diluted, plated, and colonies counted to determine bacterial survival. Phages Pumpjack, GiddyUp, Revolver, AllMyExes, and Riverwalk were all tested ($n = 3$). Bacteria only and ampicillin controls had $n = 10$. Data were analyzed with a one-way ANOVA and Dunnett's post-hoc test (bacteria only comparison), ns = not significant, (*) $p \leq 0.05$, (**) $p \leq 0.01$, (***) $p \leq 0.001$, (****) $p \leq 0.00001$. Data are shown as mean ± SD.
