# Supplementary material for: Phage mediated growth inhibition and biofilm disruption of the endodontic pathogen Enterococcus faecalis
Source: PLoS One. 2026 Jun 17;21(6):e0350657. doi: 10.1371/journal.pone.0350657 (PMC13495677; doi:10.1371/journal.pone.0350657)
Supplement: Supplementary file 2 [file pone.0350657.s003.pdf]

S2 Table. Colony Forming Unit Counts of Phage Mediated Inhibition of 4082.

Treatment	CFU/ml ± SD	% Change vs. Bacteria	Significance
Bacteria Only (4082)	2.81E+09 ± 8.85E+08	X	N/A
AllMyExes MOI (10)	4.21E+08 ± 1.13E+08	-85.0	****
AllMyExes MOI (1)	5.84E+08 ± 1.87E+08	-79.2	***
AllMyExes MOI (0.1)	4.70E+08 ± 1.62E+08	-83.2	****
AllMyExes MOI (0.01)	2.09E+08 ± 1.34E+08	-92.5	****
AllMyExes MOI (0.001)	1.48E+08 ± 1.73E+08	-94.7	****
AllMyExes MOI (0.0001)	6.78E+08 ± 9.75E+08	-75.8	***
AllMyExes MOI (0.00001)	1.33E+09 ± 9.27E+08	-52.6	*
Riverwalk MOI (10)	4.33E+08 ± 1.36E+08	-84.6	****
Riverwalk MOI (1)	4.81E+08 ± 4.06E+08	-82.9	****
Riverwalk MOI (0.1)	3.79E+08 ± 2.24E+08	-86.5	****
Riverwalk MOI (0.01)	2.61E+08 ± 3.22E+08	-90.7	****
Riverwalk MOI (0.001)	2.05E+08 ± 2.09E+08	-92.7	****
Riverwalk MOI (0.0001)	2.29E+08 ± 2.13E+08	-91.8	****
Riverwalk MOI (0.00001)	1.13E+09 ± 9.69E+08	-59.8	*
Revolver MOI (10)	9.20E+08 ± 4.26E+08	-67.2	**
Revolver MOI (1)	9.78E+08 ± 1.35E+08	-65.2	**
Revolver MOI (0.1)	1.49E+07 ± 1.83E+07	-99.5	****
Revolver MOI (0.01)	3.41E+06 ±1.95E+06	-99.9	****
Revolver MOI (0.001)	1.86E+08 ± 5.01E+07	-93.4	****
Revolver MOI (0.0001)	1.67E+09 ± 1.80E+08	-40.6	ns
Revolver MOI (0.00001)	1.90E+09 ± 2.36E+08	-32.3	ns
PricklyPear MOI (10)	1.18E+09 ± 5.41E+08	-58.0	*
PricklyPear MOI (1)	1.17E+09 ± 1.34E+09	-58.4	*
PricklyPear MOI (0.1)	1.22E+09 ± 8.78E+08	-56.5	*
PricklyPear MOI (0.01)	1.64E+09 ± 1.35E+09	-41.5	ns
PricklyPear MOI (0.001)	1.70E+09 ± 1.52E+09	-39.4	ns
PricklyPear MOI (0.0001)	9.11E+08 ± 8.56E+08	-67.6	**
PricklyPear MOI (0.00001)	1.29E+09 ± 2.23E+09	-54.0	*
TexasRanger MOI (10)	1.02E+09 ± 7.27E+08	-63.7	**
TexasRanger MOI (1)	1.80E+09 ± 7.55E+08	-35.8	ns

TexasRanger MOI (0.1)	2.41E+09 ± 2.98E+08	-14.0	ns
TexasRanger MOI (0.01)	2.09E+09 ± 3.77E+08	-25.5	ns
TexasRanger MOI (0.001)	2.41E+09 ± 4.55E+08	-14.1	ns
TexasRanger MOI (0.0001)	2.55E+09 ± 6.81E+07	-9.3	ns
TexasRanger MOI (0.00001)	2.43E+09 ± 1.88E+08	-13.3	ns
Haystack MOI (10)	1.45E+08 ± 2.14E+07	-94.8	****
Haystack MOI (1)	1.02E+09 ± 6.22E+08	-63.6	**
Haystack MOI (0.1)	1.81E+09 ± 5.74E+08	-35.5	ns
Haystack MOI (0.01)	2.52E+09 ± 5.97E+08	-10.1	ns
Haystack MOI (0.001)	2.29E+09 ± 4.35E+08	-18.5	ns
Haystack MOI (0.0001)	2.18E+09 ± 3.65E+08	-22.4	ns
Haystack MOI (0.00001)	2.38E+09 ± 1.86E+08	-15.3	ns
Stockyards MOI (10)	1.89E+09 ± 5.52E+08	-32.8	ns
Stockyards MOI (1)	1.83E+09 ± 3.15E+08	-34.7	ns
Stockyards MOI (0.1)	2.02E+09 ± 4.02E+08	-27.9	ns
Stockyards MOI (0.01)	2.00E+09 ± 3.16E+08	-28.7	ns
Stockyards MOI (0.001)	2.40E+09 ± 3.82E+08	-14.5	ns
Stockyards MOI (0.0001)	3.09E+09 ± 5.34E+08	10.0	ns
Stockyards MOI (0.00001)	2.51E+09 ± 4.37E+08	-10.5	ns
RioGrande MOI (10)	1.37E+09 ± 2.31E+08	-51.3	ns
RioGrande MOI (1)	1.55E+09 ± 6.34E+08	-44.6	ns
RioGrande MOI (0.1)	1.98E+09 ± 9.33E+08	-29.5	ns
RioGrande MOI (0.01)	2.43E+09 ± 1.39E+09	-13.3	ns
RioGrande MOI (0.001)	2.38E+09 ± 8.87E+08	-15.3	ns
RioGrande MOI (0.0001)	3.37E+09 ± 4.04E+08	20.0	ns
RioGrande MOI (0.00001)	2.32E+09 ± 1.21E+09	-17.3	ns
Ampicillin 10 µg/ml	9.16E+06 ± 1.56E+07	-99.7	****

A total of 1.1×10^6 CFUs of bacteria were mixed with several amounts of phage ranging from 1.1×10^7 PFU to 11 PFU making multiplicities of infection (MOI) 10-0.00001. After 21 hours of growth curve testing, samples were serially diluted, plated, and colonies counted to determine bacterial survival. Phages AllMyExes, Riverwalk, Revolver, PricklyPear, TexasRanger, Haystack, Stockyards, and RioGrande were all tested (n = 3). Bacteria only and ampicillin controls had n = 9. Data were analyzed with a one-way ANOVA and Dunnett's post-hoc test (bacteria only comparison), ns = not significant, (*) $p \leq 0.05$, (**) $p \leq 0.01$, (***) $p \leq 0.001$, (****) $p \leq 0.00001$.
