# Supplementary material for: Phage mediated growth inhibition and biofilm disruption of the endodontic pathogen Enterococcus faecalis
Source: PLoS One. 2026 Jun 17;21(6):e0350657. doi: 10.1371/journal.pone.0350657 (PMC13495677; doi:10.1371/journal.pone.0350657)
Supplement: Supplementary file 3 [file pone.0350657.s008.docx]

**Journal Requirements:**

When submitting your revision, we need you to address these additional requirements.

1. Please ensure that your manuscript meets PLOS ONE's style requirements, including those for file naming. The PLOS ONE style templates can be found at

<https://journals.plos.org/plosone/s/file?id=wjVg/PLOSOne_formatting_sample_main_body.pdf> and

<https://journals.plos.org/plosone/s/file?id=ba62/PLOSOne_formatting_sample_title_authors_affiliations.pdf>

2. Please note that funding information should not appear in any section or other areas of your manuscript. We will only publish funding information present in the Funding Statement section of the online submission form. Please remove any funding-related text from the manuscript.

3. We note that your Data Availability Statement is currently as follows:

“All relevant data are within the manuscript and its Supporting Information files.”

Please confirm at this time whether or not your submission contains all raw data required to replicate the results of your study. Authors must share the “minimal data set” for their submission. PLOS defines the minimal data set to consist of the data required to replicate all study findings reported in the article, as well as related metadata and methods (<https://journals.plos.org/plosone/s/data-availability#loc-minimal-data-set-definition>).

For example, authors should submit the following data:

- The values behind the means, standard deviations and other measures reported;

- The values used to build graphs;

- The points extracted from images for analysis.

Authors do not need to submit their entire data set if only a portion of the data was used in the reported study.

If your submission does not contain these data, please either upload them as Supporting Information files or deposit them to a stable, public repository and provide us with the relevant URLs, DOIs, or accession numbers. For a list of recommended repositories, please see <https://journals.plos.org/plosone/s/recommended-repositories>.

If there are ethical or legal restrictions on sharing a de-identified data set, please explain them in detail (e.g., data contain potentially sensitive information, data are owned by a third-party organization, etc.) and who has imposed them (e.g., an ethics committee). Please also provide contact information for a data access committee, ethics committee, or other institutional body to which data requests may be sent. If data are owned by a third party, please indicate how others may request data access.

Response: Yes, we confirm that our submission contains all raw data required to replicate the results of our study.

4. We notice that your supplementary figures are included in the manuscript file. Please remove them and upload them with the file type 'Supporting Information'. Please ensure that each Supporting Information file has a legend listed in the manuscript after the references list.

5. If the reviewer comments include a recommendation to cite specific previously published works, please review and evaluate these publications to determine whether they are relevant and should be cited. There is no requirement to cite these works unless the editor has indicated otherwise. 


[Note: HTML markup is below. Please do not edit.]

Reviewers' comments:

Reviewer's Responses to Questions

**Comments to the Author**

1. Is the manuscript technically sound, and do the data support the conclusions?

The manuscript must describe a technically sound piece of scientific research with data that supports the conclusions. Experiments must have been conducted rigorously, with appropriate controls, replication, and sample sizes. The conclusions must be drawn appropriately based on the data presented.

Reviewer #1: Yes

Reviewer #2: Partly

2. Has the statistical analysis been performed appropriately and rigorously?

Reviewer #1: Yes

Reviewer #2: Yes

3. Have the authors made all data underlying the findings in their manuscript fully available?

The [PLOS Data policy](http://www.plosone.org/static/policies.action#sharing) requires authors to make all data underlying the findings described in their manuscript fully available without restriction, with rare exception (please refer to the Data Availability Statement in the manuscript PDF file). The data should be provided as part of the manuscript or its supporting information, or deposited to a public repository. For example, in addition to summary statistics, the data points behind means, medians and variance measures should be available. If there are restrictions on publicly sharing data—e.g. participant privacy or use of data from a third party—those must be specified.

Reviewer #1: Yes

Reviewer #2: Yes

4. Is the manuscript presented in an intelligible fashion and written in standard English?

PLOS ONE does not copyedit accepted manuscripts, so the language in submitted articles must be clear, correct, and unambiguous. Any typographical or grammatical errors should be corrected at revision, so please note any specific errors here.

Reviewer #1: Yes

Reviewer #2: Yes

5. Review Comments to the Author

Please use the space provided to explain your answers to the questions above. You may also include additional comments for the author, including concerns about dual publication, research ethics, or publication ethics. (Please upload your review as an attachment if it exceeds 20,000 characters)

**Reviewer #1:**

**Page 10:** Please remove the link within the text and replace with appropriate citation style.

Response: Link was removed and cited appropriately. A second link was also identified and corrected. Lines 42 and 86

**Page 11:** Please revise the Last paragraph of the introduction section as it contains results or findings of the study. The introduction should only list aim of work or objectives without findings.

Response: Results and findings in the last paragraph of the introduction have been removed. Lines 77-81

**In Table 3:** Authors should replace zero results with being below the lower limit of detection.
PH stability assays have not been performed and this is considered specifically important in the environment of endodontic infections.

Response: The phrase “Below Limit of Detection” has replaced the zero results. This table along with another have also been replaced and moved to supporting information per recommendation by the other reviewer. S1 Table, S2 Table

**Authors** should discuss clinical delivery challenges of phage therapy in endodontics and should also refer to recent studies addressing the same topic.

Response: The current discussion on this topic has been expanded as seen in Lines 213-228.

**Authors** should refer to the required future steps for this work referring to similar studies and including the importance of testing phage cocktails and the importance of testing compatibility of use with dental materials. These are considered major drawbacks for clinical translation of research. The manuscript provides phage characterization rather than testing phage performance under realistic endodontic conditions as it lacks testing stability and activity in the presence of common irrigants or endodontic material.

Response: The current discussion on this topic has been expanded as seen in Lines 197-206 and 229-240.
------------------------------------------------------------------------------------------------------------------------------

**Reviewer #2:** This manuscript by Arens et al. described a set of bacteriophages with activity against Enterococcus faecalis, which can cause endodontic infections. Phage therapy has potential for treatment of infected root canals and other type of infections, so characterizing phage activity against E. faecalis isolates is necessary for the development of phage therapy approaches. The authors test a panel of previously isolated phages against a panel of 21 E. faecalis and E. faecium isolates. They measure reduction in viability for E. faecalis target strains and disruption of biofilm. In general, I thought the experiments were carried out in a rigorous manner with proper controls and statistics. I have the following comments for consideration:

**1)** How were the E. faecalis/E. faecium target strains chosen? Rationale is given for OG1RF and strain 4082, and the ATCC strains were presumably chosen because they are the ATCC isolates. Were the VRE strains chosen at random? Or were they chosen because they belong to different sequence types/have diversity of plasmids or other genomic content?

Response: Overall, the strains were chosen based on having wide genetic variability. It was also suggested at one point while planning this initial work that because E. faecalis is present if not more prevalent in other infections it would be important to include strains that represent different infections, such as VRE to address the interests of readers broadly. The following was added to the table caption and the material and methods section, “A wide range of strains with various genetic differences are included to highlight the broad applicability of these phage, such as VRE strains which are common in hospital settings and pose a significant health risk but are not commonly associated with endodontic infections. Testing a wide range also highlights which phage are more useful in targeted versus broad spectrum treatments.” Table 2 caption, Lines 263-267

**2)** In my opinion, it would be helpful to see the individual plots for each phage-bacteria combination (Supplementary Figures 2 & 3) in the main text with the summary tables (Table 3, Table 4) in the supplement. The individual plots are helpful for the reader in that they can be used to evaluate variability across replicates.

Response: The supplemental figures have been exchanged for the tables according to the recommendation. Figures 3 and 5, Tables S1 and S2

**3)** What do the empty/blank wells mean in Table 2? No phage infection detected, or not tested?

Response: Blank wells represent no phage infection detected. A sentence was added to Table 2’s caption clarifying this point.

**4)** Biofilm formation and disruption of pre-formed biofilms was measured by OD595 staining with crystal violet, and bacterial growth (OD600 or CFU) was not measured in biofilm assays. This is an important consideration for biofilm assays as debris from dead cells might still be associated with the biofilm and thus stained with crystal violet.

Response: Completely correct, we have added a few sentences to highlight this limitation and state the incorporation of live/dead staining and CFU counts into future experiments. Here is the text for quick reference, “It is important to note that biofilm disruption was quantified only with crystal violet staining, which only measures total biomass and cannot differentiate between live and dead cells. Future experiments in more biologically relevant models, accounting for more variables, such as ex vivo teeth and potential animal work will incorporate live/dead staining and CFU counts.” Lines 197-201

**5)** The authors relate the biological relevance of the study to a potential use of phage therapy to treat enterococcal endodontic infections, but all assays done in this study used TSB. I am not necessarily suggesting that the authors should repeat all the assays in a different condition, but the phage might not be active in something like saliva (or even serum as a model for other infection sites). It is challenging to make the jump to translation of these phages for treating infections if they are not active against E. faecalis in conditions that are relevant in vivo. This is something that could be discussed as a limitation of the study.

Response: We have added the following text to the discussion in the same area as the previous comment to address this point, “In relation to biologically relevant variables, a clearer understanding of phage efficacy in saliva or other oral suspensions will be critical. This work was performed in tryptic soy broth which differs greatly in composition from saliva and endodontic spaces, which may result in deactivated phage compared to lab formulated media. A better understanding in this area will aid the transition from benchtop to clinical application.” Lines 201-206

Minor comments:

**1)** It would be helpful to combine Table 2 and Table 5 so that the source of each strain is noted next to the phage infection data. Readers may not know that the numerical strain names all correspond to ATCC strains.

Response: This is a point well made, we tried out a version of these tables combined and adding the source makes, at least in our opinion, the table a little too busy and pulls attention away from the data. However, we think it would be best to have future readers rely on the caption to gather this information (first and second sentences).

6. PLOS authors have the option to publish the peer review history of their article ([what does this mean?](https://journals.plos.org/plosone/s/editorial-and-peer-review-process#loc-peer-review-history)). If published, this will include your full peer review and any attached files.

If you choose “no”, your identity will remain anonymous but your review may still be made public.

**Do you want your identity to be public for this peer review?** For information about this choice, including consent withdrawal, please see our [Privacy Policy](https://www.plos.org/privacy-policy).

Reviewer #1: No

Reviewer #2: No
