# Supplementary material for: Phage mediated growth inhibition and biofilm disruption of the endodontic pathogen Enterococcus faecalis
Source: PLoS One. 2026 Jun 17;21(6):e0350657. doi: 10.1371/journal.pone.0350657 (PMC13495677; doi:10.1371/journal.pone.0350657)
Supplement: Supplementary file 4 [file pone.0350657.s009.docx]

PONE-D-26-02348R1

Phage Mediated Growth Inhibition and Biofilm Disruption of the Endodontic Pathogen Enterococcus faecalis

PLOS One

Additional Editor Comments:

1. "pH stability assays have not been performed." Why?

**Response:** We took the approach that while pH stability is important, to test it outside the context of endodontic sealers would detract from the focus of the paper. Planned future studies that involve the combination of phage and sealers will have pH monitored. The following sentence was added at lines 260-262. “Phage pH dependent stability, not tested in this investigation, will be tested in conjunction with sealers as it will be more clinically relevant to this application than pH testing alone.”

2. An explanation has been added regarding "wide genetic variability," but the following elements are still lacking:

- Genomic characterization
- Sequence types (MLST)
- Resistance profiles beyond the VRE label

**Response:** The following sentences and website link were added at lines 295-299. **“**All genomic characterization, accession numbers, and resistance profiles are available on the AR Isolate Bank website (<https://wwwn.cdc.gov/ARIsolateBank/>) while accession numbers and resistance mechanisms can be found in Table 3 . Isolates were identified by the CDC using Matrix-Assisted Laser Desorption Ionization-Time of Flight Mass Spectrometry (MALDI-TOF MS).” Additionally, Table 3 has been altered to include Biosample Accession numbers and resistance mechanisms of the CDC strains for ease of access.

3. Additionally, some inconsistencies remain. This approach is unusual and may introduce bias.

- Different n values across experiments
  - (e.g., n=9, n=10, n=11 for controls)
- CFU counts are derived from: "median OD wells"

**Response:** Lines in 351-363 have been added explaining that nine and eleven plates were needed to test all of the phage against the two strains and because controls were included on each assay plate to account for inter-plate variability the replicate numbers were higher. The replicate number of ten is due to damage of agar plates before counting could be performed and has been explained.

Further explanation of median OD wells being taken from technical replicates and not biological replicates is provided.

4. MOI-Dependent Inconsistencies Not Explained

- Some phages perform worse at higher MOI
- Some exhibit non-linear effects

**Response:** A discussion of these results can be found in lines 195-217.

5. No mechanistic explanations have been provided for:

- Phage adsorption
- Resistance emergence
- Lysis inhibition

**Response:** Lysis inhibition is discussed in in lines 195-217 (noted above). Phage adsorption is mentioned in lines 232-235 while resistance emergence is in lines 262-265. Both adsorption and resistance are not discussed thoroughly in an effort to keep the manuscript focused despite the topics being of great interest.

6. Overinterpretation of Results Based on In-Vitro Data Alone

Examples include:

- "100% inhibition"
- "Exceptionally effective"
- "Strong therapeutic potential"

**Response:** Wording has been removed or altered in lines 31-32, 122-123, 182, 219, 239, 276. Supplementary Table 1 has also been updated to remove mentions of 100% efficacy.

[Note: HTML markup is below. Please do not edit.]

[NOTE: If reviewer comments were submitted as an attachment file, they will be attached to this email and accessible via the submission site. Please log into your account, locate the manuscript record, and check for the action link "View Attachments". If this link does not appear, there are no attachment files.]

To ensure your figures meet our technical requirements, please review our figure guidelines: https://journals.plos.org/plosone/s/figures

You may also use PLOS’s free figure tool, NAAS, to help you prepare publication quality figures: https://journals.plos.org/plosone/s/figures#loc-tools-for-figure-preparation.

NAAS will assess whether your figures meet our technical requirements by comparing each figure against our figure specifications.
