# Supplementary material for: Phage mediated growth inhibition and biofilm disruption of the endodontic pathogen Enterococcus faecalis
Source: PLoS One. 2026 Jun 17;21(6):e0350657. doi: 10.1371/journal.pone.0350657 (PMC13495677; doi:10.1371/journal.pone.0350657)
Supplement: Peer review history

# Peer review: Decision Letter 0

**DOI:** 10.1371/journal.pone.0350657.r001

**Authors:** Md Bashir Uddin

9 Mar 2026

PONE-D-26-02348

Phage Mediated Growth Inhibition and Biofilm Disruption of the Endodontic ;Pathogen Enterococcus faecalis

PLOS One

Dear Dr. Hwang,

Thank you for submitting your manuscript to PLOS ONE. After careful consideration, we feel that it has merit but does not fully meet PLOS ONE’s publication criteria as it currently stands. Therefore, we invite you to submit a revised version of the manuscript that addresses the points raised during the review process.

Please submit your revised manuscript by Apr 23 2026 11:59PM. If you will need more time than this to complete your revisions, please reply to this message or contact the journal office at plosone@plos.org. When you're ready to submit your revision, log on to https://www.editorialmanager.com/pone/ and select the 'Submissions Needing Revision' folder to locate your manuscript file.

Please include the following items when submitting your revised manuscript:

- A letter that responds to each point raised by the academic editor and reviewer(s). You should upload this letter as a separate file labeled 'Response to Reviewers'.
- A marked-up copy of your manuscript that highlights changes made to the original version. You should upload this as a separate file labeled 'Revised Manuscript with Track Changes'.
- An unmarked version of your revised paper without tracked changes. You should upload this as a separate file labeled 'Manuscript'.

If you would like to make changes to your financial disclosure, please include your updated statement in your cover letter. Guidelines for resubmitting your figure files are available below the reviewer comments at the end of this letter.

If applicable, we recommend that you deposit your laboratory protocols in protocols.io to enhance the reproducibility of your results. Protocols.io assigns your protocol its own identifier (DOI) so that it can be cited independently in the future. For instructions see: https://journals.plos.org/plosone/s/submission-guidelines#loc-laboratory-protocols. Additionally, PLOS ONE offers an option for publishing peer-reviewed Lab Protocol articles, which describe protocols hosted on protocols.io. Read more information on sharing protocols at https://plos.org/protocols?utm_medium=editorial-email&utm_source=authorletters&utm_campaign=protocols.

We look forward to receiving your revised manuscript.

Kind regards,

Md Bashir Uddin, PhD

Academic Editor

PLOS One

**Journal Requirements:** 

When submitting your revision, we need you to address these additional requirements.

1. Please ensure that your manuscript meets PLOS ONE's style requirements, including those for file naming. The PLOS ONE style templates can be found at

https://journals.plos.org/plosone/s/file?id=wjVg/PLOSOne_formatting_sample_main_body.pdf and

https://journals.plos.org/plosone/s/file?id=ba62/PLOSOne_formatting_sample_title_authors_affiliations.pdf

2. Please note that funding information should not appear in any section or other areas of your manuscript. We will only publish funding information present in the Funding Statement section of the online submission form. Please remove any funding-related text from the manuscript.

3. We note that your Data Availability Statement is currently as follows:

“All relevant data are within the manuscript and its Supporting Information files.”

Please confirm at this time whether or not your submission contains all raw data required to replicate the results of your study. Authors must share the “minimal data set” for their submission. PLOS defines the minimal data set to consist of the data required to replicate all study findings reported in the article, as well as related metadata and methods (https://journals.plos.org/plosone/s/data-availability#loc-minimal-data-set-definition).

For example, authors should submit the following data:

- The values behind the means, standard deviations and other measures reported;

- The values used to build graphs;

- The points extracted from images for analysis.

Authors do not need to submit their entire data set if only a portion of the data was used in the reported study.

If your submission does not contain these data, please either upload them as Supporting Information files or deposit them to a stable, public repository and provide us with the relevant URLs, DOIs, or accession numbers. For a list of recommended repositories, please see https://journals.plos.org/plosone/s/recommended-repositories.

If there are ethical or legal restrictions on sharing a de-identified data set, please explain them in detail (e.g., data contain potentially sensitive information, data are owned by a third-party organization, etc.) and who has imposed them (e.g., an ethics committee). Please also provide contact information for a data access committee, ethics committee, or other institutional body to which data requests may be sent. If data are owned by a third party, please indicate how others may request data access.

4. We notice that your supplementary figures are included in the manuscript file. Please remove them and upload them with the file type 'Supporting Information'. Please ensure that each Supporting Information file has a legend listed in the manuscript after the references list.

5. If the reviewer comments include a recommendation to cite specific previously published works, please review and evaluate these publications to determine whether they are relevant and should be cited. There is no requirement to cite these works unless the editor has indicated otherwise.

[Note: HTML markup is below. Please do not edit.]

Reviewers' comments:

Reviewer's Responses to Questions

**Comments to the Author** 

1. Is the manuscript technically sound, and do the data support the conclusions?

The manuscript must describe a technically sound piece of scientific research with data that supports the conclusions. Experiments must have been conducted rigorously, with appropriate controls, replication, and sample sizes. The conclusions must be drawn appropriately based on the data presented.

Reviewer #1: Yes

Reviewer #2: Partly

**********

2. Has the statistical analysis been performed appropriately and rigorously?

Reviewer #1: Yes

Reviewer #2: Yes

**********

3. Have the authors made all data underlying the findings in their manuscript fully available?

The PLOS Data policy requires authors to make all data underlying the findings described in their manuscript fully available without restriction, with rare exception (please refer to the Data Availability Statement in the manuscript PDF file). The data should be provided as part of the manuscript or its supporting information, or deposited to a public repository. For example, in addition to summary statistics, the data points behind means, medians and variance measures should be available. If there are restrictions on publicly sharing data—e.g. participant privacy or use of data from a third party—those must be specified.

Reviewer #1: Yes

Reviewer #2: Yes

**********

4. Is the manuscript presented in an intelligible fashion and written in standard English?

PLOS ONE does not copyedit accepted manuscripts, so the language in submitted articles must be clear, correct, and unambiguous. Any typographical or grammatical errors should be corrected at revision, so please note any specific errors here.

Reviewer #1: Yes

Reviewer #2: Yes

**********

5. Review Comments to the Author

Please use the space provided to explain your answers to the questions above. You may also include additional comments for the author, including concerns about dual publication, research ethics, or publication ethics. (Please upload your review as an attachment if it exceeds 20,000 characters)

Reviewer #1: Page 10: Please remove the link within the text and replace with appropriate citation style.

Page 11: Please revise the Last paragraph of the introduction section as it contains results or findings of the study. The introduction should only list aim of work or objectives without findings.

In Table 3: Authors should replace zero results with being below the lower limit of detection.

PH stability assays have not been performed and this is considered specifically important in the environment of endodontic infections.

Authors should discuss clinical delivery challenges of phage therapy in endodontics and should also refer to recent studies addressing the same topic.

Authors should refer to the required future steps for this work referring to similar studies and including the importance of testing phage cocktails and the importance of testing compatibility of use with dental materials. These are considered major drawbacks for clinical translation of research.

The manuscript provides phage characterization rather than testing phage performance under realistic endodontic conditions as it lacks testing stability and activity in the presence of common irrigants or endodontic material.

Reviewer #2: This manuscript by Arens et al. described a set of bacteriophages with activity against Enterococcus faecalis, which can cause endodontic infections. Phage therapy has potential for treatment of infected root canals and other type of infections, so characterizing phage activity against E. faecalis isolates is necessary for the development of phage therapy approaches. The authors test a panel of previously isolated phages against a panel of 21 E. faecalis and E. faecium isolates. They measure reduction in viability for E. faecalis target strains and disruption of biofilm. In general, I thought the experiments were carried out in a rigorous manner with proper controls and statistics. I have the following comments for consideration:

1) How were the E. faecalis/E. faecium target strains chosen? Rationale is given for OG1RF and strain 4082, and the ATCC strains were presumably chosen because they are the ATCC isolates. Were the VRE strains chosen at random? Or were they chosen because they belong to different sequence types/have diversity of plasmids or other genomic content?

2) In my opinion, it would be helpful to see the individual plots for each phage-bacteria combination (Supplementary Figures 2 & 3) in the main text with the summary tables (Table 3, Table 4) in the supplement. The individual plots are helpful for the reader in that they can be used to evaluate variability across replicates.

3) What do the empty/blank wells mean in Table 2? No phage infection detected, or not tested?

4) Biofilm formation and disruption of pre-formed biofilms was measured by OD595 staining with crystal violet, and bacterial growth (OD600 or CFU) was not measured in biofilm assays. This is an important consideration for biofilm assays as debris from dead cells might still be associated with the biofilm and thus stained with crystal violet.

5) The authors relate the biological relevance of the study to a potential use of phage therapy to treat enterococcal endodontic infections, but all assays done in this study used TSB. I am not necessarily suggesting that the authors should repeat all the assays in a different condition, but the phage might not be active in something like saliva (or even serum as a model for other infection sites). It is challenging to make the jump to translation of these phages for treating infections if they are not active against E. faecalis in conditions that are relevant in vivo. This is something that could be discussed as a limitation of the study.

Minor comments:

1) It would be helpful to combine Table 2 and Table 5 so that the source of each strain is noted next to the phage infection data. Readers may not know that the numerical strain names all correspond to ATCC strains.

**********

6. PLOS authors have the option to publish the peer review history of their article (what does this mean?). If published, this will include your full peer review and any attached files.

If you choose “no”, your identity will remain anonymous but your review may still be made public.

**Do you want your identity to be public for this peer review?**    For information about this choice, including consent withdrawal, please see our Privacy Policy.

Reviewer #1: No

Reviewer #2: No

**********

[NOTE: If reviewer comments were submitted as an attachment file, they will be attached to this email and accessible via the submission site. Please log into your account, locate the manuscript record, and check for the action link "View Attachments". If this link does not appear, there are no attachment files.]

To ensure your figures meet our technical requirements, please review our figure guidelines: https://journals.plos.org/plosone/s/figures

You may also use PLOS’s free figure tool, NAAS, to help you prepare publication quality figures: https://journals.plos.org/plosone/s/figures#loc-tools-for-figure-preparation.

NAAS will assess whether your figures meet our technical requirements by comparing each figure against our figure specifications.

# Author comment: Author response to Decision Letter 1

**DOI:** 10.1371/journal.pone.0350657.r002

15 Apr 2026

We confirm that all data relevant to replicating this study has been included.

Reviewer #1:

Page 10: Please remove the link within the text and replace with appropriate citation style.

Response: Link was removed and cited appropriately. A second link was also identified and corrected. Lines 42 and 86

Page 11: Please revise the Last paragraph of the introduction section as it contains results or findings of the study. The introduction should only list aim of work or objectives without findings.

Response: Results and findings in the last paragraph of the introduction have been removed. Lines 77-81

In Table 3: Authors should replace zero results with being below the lower limit of detection.

PH stability assays have not been performed and this is considered specifically important in the environment of endodontic infections.

Response: The phrase “Below Limit of Detection” has replaced the zero results. This table along with another have also been replaced and moved to supporting information per recommendation by the other reviewer. S1 Table, S2 Table

Authors should discuss clinical delivery challenges of phage therapy in endodontics and should also refer to recent studies addressing the same topic.

Response: The current discussion on this topic has been expanded as seen in Lines 213-228.

Authors should refer to the required future steps for this work referring to similar studies and including the importance of testing phage cocktails and the importance of testing compatibility of use with dental materials. These are considered major drawbacks for clinical translation of research. The manuscript provides phage characterization rather than testing phage performance under realistic endodontic conditions as it lacks testing stability and activity in the presence of common irrigants or endodontic material.

Response: The current discussion on this topic has been expanded as seen in Lines 197-206 and 229-240.

------------------------------------------------------------------------------------------------------------------------------

Reviewer #2: This manuscript by Arens et al. described a set of bacteriophages with activity against Enterococcus faecalis, which can cause endodontic infections. Phage therapy has potential for treatment of infected root canals and other type of infections, so characterizing phage activity against E. faecalis isolates is necessary for the development of phage therapy approaches. The authors test a panel of previously isolated phages against a panel of 21 E. faecalis and E. faecium isolates. They measure reduction in viability for E. faecalis target strains and disruption of biofilm. In general, I thought the experiments were carried out in a rigorous manner with proper controls and statistics. I have the following comments for consideration:

1) How were the E. faecalis/E. faecium target strains chosen? Rationale is given for OG1RF and strain 4082, and the ATCC strains were presumably chosen because they are the ATCC isolates. Were the VRE strains chosen at random? Or were they chosen because they belong to different sequence types/have diversity of plasmids or other genomic content?

Response: Overall, the strains were chosen based on having wide genetic variability. It was also suggested at one point while planning this initial work that because E. faecalis is present if not more prevalent in other infections it would be important to include strains that represent different infections, such as VRE to address the interests of readers broadly. The following was added to the table caption and the material and methods section, “A wide range of strains with various genetic differences are included to highlight the broad applicability of these phage, such as VRE strains which are common in hospital settings and pose a significant health risk but are not commonly associated with endodontic infections. Testing a wide range also highlights which phage are more useful in targeted versus broad spectrum treatments.” Table 2 caption, Lines 263-267

2) In my opinion, it would be helpful to see the individual plots for each phage-bacteria combination (Supplementary Figures 2 & 3) in the main text with the summary tables (Table 3, Table 4) in the supplement. The individual plots are helpful for the reader in that they can be used to evaluate variability across replicates.

Response: The supplemental figures have been exchanged for the tables according to the recommendation. Figures 3 and 5, Tables S1 and S2

3) What do the empty/blank wells mean in Table 2? No phage infection detected, or not tested?

Response: Blank wells represent no phage infection detected. A sentence was added to Table 2’s caption clarifying this point.

4) Biofilm formation and disruption of pre-formed biofilms was measured by OD595 staining with crystal violet, and bacterial growth (OD600 or CFU) was not measured in biofilm assays. This is an important consideration for biofilm assays as debris from dead cells might still be associated with the biofilm and thus stained with crystal violet.

Response: Completely correct, we have added a few sentences to highlight this limitation and state the incorporation of live/dead staining and CFU counts into future experiments. Here is the text for quick reference, “It is important to note that biofilm disruption was quantified only with crystal violet staining, which only measures total biomass and cannot differentiate between live and dead cells. Future experiments in more biologically relevant models, accounting for more variables, such as ex vivo teeth and potential animal work will incorporate live/dead staining and CFU counts.” Lines 197-201

5) The authors relate the biological relevance of the study to a potential use of phage therapy to treat enterococcal endodontic infections, but all assays done in this study used TSB. I am not necessarily suggesting that the authors should repeat all the assays in a different condition, but the phage might not be active in something like saliva (or even serum as a model for other infection sites). It is challenging to make the jump to translation of these phages for treating infections if they are not active against E. faecalis in conditions that are relevant in vivo. This is something that could be discussed as a limitation of the study.

Response: We have added the following text to the discussion in the same area as the previous comment to address this point, “In relation to biologically relevant variables, a clearer understanding of phage efficacy in saliva or other oral suspensions will be critical. This work was performed in tryptic soy broth which differs greatly in composition from saliva and endodontic spaces, which may result in deactivated phage compared to lab formulated media. A better understanding in this area will aid the transition from benchtop to clinical application.” Lines 201-206

Minor comments:

1) It would be helpful to combine Table 2 and Table 5 so that the source of each strain is noted next to the phage infection data. Readers may not know that the numerical strain names all correspond to ATCC strains.

Response: This is a point well made, we tried out a version of these tables combined and adding the source makes, at least in our opinion, the table a little too busy and pulls attention away from the data. However, we think it would be best to have future readers rely on the caption to gather this information (first and second sentences).

Attachment Submitted filename: Response to Reviewers.docx

# Editor report: Decision Letter 1

**DOI:** 10.1371/journal.pone.0350657.r003

**Authors:** Md Bashir Uddin

30 Apr 2026

PONE-D-26-02348R1

Phage Mediated Growth Inhibition and Biofilm Disruption of the Endodontic Pathogen Enterococcus faecalis

PLOS One

Dear Dr.  Hwang,

Thank you for submitting your manuscript to PLOS ONE. After careful consideration, we feel that it has merit but does not fully meet PLOS ONE’s publication criteria as it currently stands. Therefore, we invite you to submit a revised version of the manuscript that addresses the points raised during the review process.

Please submit your revised manuscript by Jun 14 2026 11:59PM. If you will need more time than this to complete your revisions, please reply to this message or contact the journal office at plosone@plos.org. When you're ready to submit your revision, log on to https://www.editorialmanager.com/pone/ and select the 'Submissions Needing Revision' folder to locate your manuscript file.

Please include the following items when submitting your revised manuscript:

- A letter that responds to each point raised by the academic editor and reviewer(s). You should upload this letter as a separate file labeled 'Response to Reviewers'.
- A marked-up copy of your manuscript that highlights changes made to the original version. You should upload this as a separate file labeled 'Revised Manuscript with Track Changes'.
- An unmarked version of your revised paper without tracked changes. You should upload this as a separate file labeled 'Manuscript'.

If you would like to make changes to your financial disclosure, please include your updated statement in your cover letter. Guidelines for resubmitting your figure files are available below the reviewer comments at the end of this letter.

If applicable, we recommend that you deposit your laboratory protocols in protocols.io to enhance the reproducibility of your results. Protocols.io assigns your protocol its own identifier (DOI) so that it can be cited independently in the future. For instructions see: https://journals.plos.org/plosone/s/submission-guidelines#loc-laboratory-protocols. Additionally, PLOS ONE offers an option for publishing peer-reviewed Lab Protocol articles, which describe protocols hosted on protocols.io. Read more information on sharing protocols at https://plos.org/protocols?utm_medium=editorial-email&utm_source=authorletters&utm_campaign=protocols.

As the corresponding author, your ORCID iD is verified in the submission system and will appear in the published article. PLOS supports the use of ORCID, and we encourage all coauthors to register for an ORCID iD and use it as well. Please encourage your coauthors to verify their ORCID iD within the submission system before final acceptance, as unverified ORCID iDs will not appear in the published article. *Only*    the individual author can complete the verification step; PLOS staff *cannot*    verify ORCID iDs on behalf of authors.

We look forward to receiving your revised manuscript.

Kind regards,

Md Bashir Uddin, PhD

Academic Editor

PLOS One

Journal Requirements:

1. If the reviewer comments include a recommendation to cite specific previously published works, please review and evaluate these publications to determine whether they are relevant and should be cited. There is no requirement to cite these works unless the editor has indicated otherwise.

2. Please review your reference list to ensure that it is complete and correct. If you have cited papers that have been retracted, please include the rationale for doing so in the manuscript text, or remove these references and replace them with relevant current references. Any changes to the reference list should be mentioned in the rebuttal letter that accompanies your revised manuscript. If you need to cite a retracted article, indicate the article’s retracted status in the References list and also include a citation and full reference for the retraction notice.

Additional Editor Comments:

1. "pH stability assays have not been performed." Why?

2. An explanation has been added regarding "wide genetic variability," but the following elements are still lacking:

- Genomic characterization
- Sequence types (MLST)
- Resistance profiles beyond the VRE label

3. Additionally, some inconsistencies remain. This approach is unusual and may introduce bias.

- Different n values across experiments
- (e.g., n=9, n=10, n=11 for controls)
- CFU counts are derived from: "median OD wells"
  4. MOI-Dependent Inconsistencies Not Explained
- Some phages perform worse at higher MOI
- Some exhibit non-linear effects

5. No mechanistic explanations have been provided for:

- Phage adsorption
- Resistance emergence
- Lysis inhibition

6. Overinterpretation of Results Based on In-Vitro Data Alone

Examples include:

- "100% inhibition"
- "Exceptionally effective"
- "Strong therapeutic potential"

[Note: HTML markup is below. Please do not edit.]

[NOTE: If reviewer comments were submitted as an attachment file, they will be attached to this email and accessible via the submission site. Please log into your account, locate the manuscript record, and check for the action link "View Attachments". If this link does not appear, there are no attachment files.]

To ensure your figures meet our technical requirements, please review our figure guidelines: https://journals.plos.org/plosone/s/figures

You may also use PLOS’s free figure tool, NAAS, to help you prepare publication quality figures: https://journals.plos.org/plosone/s/figures#loc-tools-for-figure-preparation.

NAAS will assess whether your figures meet our technical requirements by comparing each figure against our figure specifications.

# Author comment: Author response to Decision Letter 2

**DOI:** 10.1371/journal.pone.0350657.r004

11 May 2026

PONE-D-26-02348R1

Phage Mediated Growth Inhibition and Biofilm Disruption of the Endodontic Pathogen Enterococcus faecalis

PLOS One

Additional Editor Comments:

1. "pH stability assays have not been performed." Why?

Response: We took the approach that while pH stability is important, to test it outside the context of endodontic sealers would detract from the focus of the paper. Planned future studies that involve the combination of phage and sealers will have pH monitored. The following sentence was added at lines 260-262. “Phage pH dependent stability, not tested in this investigation, will be tested in conjunction with sealers as it will be more clinically relevant to this application than pH testing alone.”

2. An explanation has been added regarding "wide genetic variability," but the following elements are still lacking:

• Genomic characterization

• Sequence types (MLST)

• Resistance profiles beyond the VRE label

Response: The following sentences and website link were added at lines 295-299. “All genomic characterization, accession numbers, and resistance profiles are available on the AR Isolate Bank website (https://wwwn.cdc.gov/ARIsolateBank/) while accession numbers and resistance mechanisms can be found in Table 3 . Isolates were identified by the CDC using Matrix-Assisted Laser Desorption Ionization-Time of Flight Mass Spectrometry (MALDI-TOF MS).” Additionally, Table 3 has been altered to include Biosample Accession numbers and resistance mechanisms of the CDC strains for ease of access.

3. Additionally, some inconsistencies remain. This approach is unusual and may introduce bias.

• Different n values across experiments

o (e.g., n=9, n=10, n=11 for controls)

• CFU counts are derived from: "median OD wells"

Response: Lines in 351-363 have been added explaining that nine and eleven plates were needed to test all of the phage against the two strains and because controls were included on each assay plate to account for inter-plate variability the replicate numbers were higher. The replicate number of ten is due to damage of agar plates before counting could be performed and has been explained.

Further explanation of median OD wells being taken from technical replicates and not biological replicates is provided.

4. MOI-Dependent Inconsistencies Not Explained

• Some phages perform worse at higher MOI

• Some exhibit non-linear effects

Response: A discussion of these results can be found in lines 195-217.

5. No mechanistic explanations have been provided for:

• Phage adsorption

• Resistance emergence

• Lysis inhibition

Response: Lysis inhibition is discussed in in lines 195-217 (noted above). Phage adsorption is mentioned in lines 232-235 while resistance emergence is in lines 262-265. Both adsorption and resistance are not discussed thoroughly in an effort to keep the manuscript focused despite the topics being of great interest.

6. Overinterpretation of Results Based on In-Vitro Data Alone

Examples include:

• "100% inhibition"

• "Exceptionally effective"

• "Strong therapeutic potential"

Response: Wording has been removed or altered in lines 31-32, 122-123, 182, 219, 239, 276. Supplementary Table 1 has also been updated to remove mentions of 100% efficacy.

[Note: HTML markup is below. Please do not edit.]

[NOTE: If reviewer comments were submitted as an attachment file, they will be attached to this email and accessible via the submission site. Please log into your account, locate the manuscript record, and check for the action link "View Attachments". If this link does not appear, there are no attachment files.]

To ensure your figures meet our technical requirements, please review our figure guidelines: https://journals.plos.org/plosone/s/figures

You may also use PLOS’s free figure tool, NAAS, to help you prepare publication quality figures: https://journals.plos.org/plosone/s/figures#loc-tools-for-figure-preparation.

NAAS will assess whether your figures meet our technical requirements by comparing each figure against our figure specifications.

Attachment Submitted filename: Response_to_Reviewers_auresp_2.docx

# Editor report: Decision Letter 2

**DOI:** 10.1371/journal.pone.0350657.r005

**Authors:** Md Bashir Uddin

17 May 2026

Phage Mediated Growth Inhibition and Biofilm Disruption of the Endodontic Pathogen Enterococcus faecalis

PONE-D-26-02348R2

Dear Dr. Hwang,

We’re pleased to inform you that your manuscript has been judged scientifically suitable for publication and will be formally accepted for publication once it meets all outstanding technical requirements.

Within one week, you’ll receive an e-mail detailing the required amendments. When these have been addressed, you’ll receive a formal acceptance letter and your manuscript will be scheduled for publication.

An invoice will be generated when your article is formally accepted. Please note, if your institution has a publishing partnership with PLOS and your article meets the relevant criteria, all or part of your publication costs will be covered. Please make sure your user information is up-to-date by logging into Editorial Manager at Editorial Manager® and clicking the ‘Update My Information' link at the top of the page. For questions related to billing, please contact billing support.

If your institution or institutions have a press office, please notify them about your upcoming paper to help maximize its impact. If they’ll be preparing press materials, please inform our press team as soon as possible -- no later than 48 hours after receiving the formal acceptance. Your manuscript will remain under strict press embargo until 2 pm Eastern Time on the date of publication. For more information, please contact onepress@plos.org.

Kind regards,

Md Bashir Uddin, PhD

Academic Editor

PLOS One

Additional Editor Comments (optional):

Reviewers' comments:

# Editor report: Acceptance letter

**DOI:** 10.1371/journal.pone.0350657.r006

**Authors:** Md Bashir Uddin

PONE-D-26-02348R2

PLOS One

Dear Dr. Hwang,

I'm pleased to inform you that your manuscript has been deemed suitable for publication in PLOS One. Congratulations! Your manuscript is now being handed over to our production team.

At this stage, our production department will prepare your paper for publication. This includes ensuring the following:

* All references, tables, and figures are properly cited

* All relevant supporting information is included in the manuscript submission,

* There are no issues that prevent the paper from being properly typeset

You will receive further instructions from the production team, including instructions on how to review your proof when it is ready. Please keep in mind that we are working through a large volume of accepted articles, so please give us a few days to review your paper and let you know the next and final steps.

Lastly, if your institution or institutions have a press office, please let them know about your upcoming paper now to help maximize its impact. If they'll be preparing press materials, please inform our press team within the next 48 hours. Your manuscript will remain under strict press embargo until 2 pm Eastern Time on the date of publication. For more information, please contact onepress@plos.org.

You will receive an invoice from PLOS for your publication fee after your manuscript has reached the completed accept phase. If you receive an email requesting payment before acceptance or for any other service, this may be a phishing scheme. Learn how to identify phishing emails and protect your accounts at https://explore.plos.org/phishing.

If we can help with anything else, please email us at customercare@plos.org.

Thank you for submitting your work to PLOS ONE and supporting open access.

Kind regards,

PLOS ONE Editorial Office Staff

on behalf of

Dr. Md Bashir Uddin

Academic Editor

PLOS One
